# Climate-Change Projections of Habitat Suitability and Spatial Patterns for Three Giant Panda Food Bamboos in the Qionglai Mountains Region, China

**DOI:** 10.3390/plants15213408

**Published:** 2026-11-02

**Authors:** Peilong Li, Jing Wang, Peihao Peng, Weiyi Zhu, Hanyi Zhang, Juan Wang

**Affiliations:** 1 College of Geography and Planning, Chengdu University of Technology, Chengdu 610059, China; 18236340110@163.com (P.L.); wangjing66@stu.cdut.edu.cn (J.W.); pengpeihao@cdut.edu.cn (P.P.); zhuweiyi@stu.cdut.edu.cn (W.Z.); zzh1341530534@gmail.com (H.Z.); 2 Institute of Ecological Resources and Landscape, Chengdu University of Technology, Chengdu 610059, China; 3 Sichuan Provincial Forestry and Grassland Key Laboratory of Biodiversity Conservation and Sustainable Community Development in Giant Panda National Park, Chengdu 611130, China

**Keywords:** food bamboo species, climate change, species distribution modeling, plant conservation, MaxEnt, threshold sensitivity, landscape pattern, giant panda

## Abstract

Climate change may alter mountain bamboo distributions and giant panda food resources. We modeled potential habitat suitability for *Bashania faberi*, *Yushania brevipaniculata*, and *Chimonobambusa szechuanensis* in a defined Qionglai Mountains study area in Sichuan, China, using MaxEnt, ten bootstrap replicates, and three global climate models (GCMs) for the 2050s and 2070s under SSP1-2.6 and SSP5-8.5. We tested sensitivity to predictors, thresholds, spatial grain, connectivity, and future climate products. High omission in some spatial folds and 15 records make *B. faberi* projections exploratory and its patch metrics indicative. At species-specific 10th-percentile (10P) thresholds, the nine-variable ensemble projected gains for *B. faberi* and *Y. brevipaniculata* only under SSP5-8.5 in the 2070s; the five-variable configuration and fixed thresholds reversed both. Each expansion was supported by one GCM, while two projected contraction. *Chimonobambusa szechuanensis* contracted under the nine-variable and elevation-containing configurations but increased in the temperature-inclusive diagnostic at 10P. The combined suitable-area proportion fell from 34.20% currently to 21.00–31.05% in baseline future maps; mean patch area and aggregation also declined. Averaging outcomes could produce a different change direction from averaging predictions. These projections describe conditional suitability, not food supply or functional connectivity.

## 1. Introduction

Giant pandas rely heavily on bamboo, but their food choices depend on more than the presence of bamboo forests. In the wild, individuals adjust their use of bamboo species and plant parts seasonally [1]. Nutritional composition varies among species and plant parts [2,3,4], and aboveground biomass differs among species [5]. The effects of bamboo age on nutritional composition also depend on species, plant part, and nutritional indicator [3,6]. Assessing climate-related changes in bamboo distributions and their implications for potential food resources therefore requires examining both the suitable area of individual bamboo species and the spatial distribution and overlap of suitable areas across species.

The Qionglai Mountains are characterized by pronounced topographic variation, with distinct temperature, moisture, and vegetation gradients extending from low-elevation river valleys to high mountain ridges. Field studies conducted in different regions have shown that food bamboo species differ in their population characteristics, spatial distributions, and associations with local environmental conditions [7,8]. Giant panda habitat use is also closely related to elevation, bamboo forest characteristics, and forest structure [9]. Early modeling studies in the Qionglai Mountains predicted that food bamboo species and giant panda habitat might shift toward higher elevations under climate change [10]. A recent comparison of two food bamboo species, identified in that study as *Bashania fangiana* (A.Camus) Keng f. & T.H.Wen and *Yushania brevipaniculata* (Hand.-Mazz.) T.P.Yi, likewise showed that the direction and magnitude of projected changes in suitable area can differ markedly between species [11]. Therefore, comparing the climatic responses of multiple food bamboo species within the same region and under a unified modeling framework can help clarify future changes in potential food resources.

Bamboo responses to climate change vary with spatial scale and species. Shang et al. [12] projected expansion of the total area of communities comprising 20 bamboo species consumed by giant pandas in Sichuan. However, newly suitable areas were projected to have relatively low bamboo species diversity, and some areas with currently high diversity were projected to lose diversity. Conversely, Zhao et al. [13] projected a contraction of suitable area for *Bashania fargesii* (E.G.Camus) Keng f. & T.P.Yi in the Qinling Mountains, particularly under Representative Concentration Pathway (RCP) scenario RCP8.5 in the 2070s. Warming experiments also revealed nonlinear responses. Yang et al. [14], studying the species reported as *Bashania fangiana*, found population size and aboveground biomass broadly unchanged under 1.5 °C warming but reduced under 3 °C and 4.5 °C warming, alongside lower nutritional value and greater aphid infestation. Zhang et al. [15], studying *Bashania faberi* (Rendle) T.P.Yi, found that lower levels of warming promoted some soil nitrogen cycling processes and increased leaf nitrogen content and biomass, whereas stronger warming reduced survival and biomass. Community area, species-level suitability, and population performance thus capture different aspects of climate impacts. Flora of China treats *Bashania faberi* (Rendle) T.P.Yi and *B. fangiana* (A.Camus) Keng f. & T.H.Wen as synonyms of *Arundinaria faberi* Rendle [16]. We retain *B. faberi* for the modeled species and the original names when discussing the cited studies.

Local conditions and climatic extremes can further modify the responses of mountain species. Geres et al. [17] found generally upward but species-specific elevational shifts across fungi, plants, and animals; shifts toward closed-canopy environments were associated with weaker upslope shifts, suggesting microclimatic buffering. Across 29 Chinese mountains, Zu et al. [18] found that lower distribution limits shifted upslope while upper limits shifted downslope, indicating range contraction rather than uniform upslope movement. Wu et al. [19] showed that including extreme climatic events could change the projected direction of suitable-area change for alpine and subalpine *Rhododendron* species. Long-term mean climate and total suitable area alone may therefore incompletely characterize mountain plant responses.

Patch structure and spatial relationships among species also matter for potential food resources. Zhou et al. [20] used MaxEnt to project distributions and Schoener’s *D* to quantify overlap. They found a projected increase in mean overlap between *Zelkova schneideriana* Hand.-Mazz. and *Celtis sinensis* Pers. but a decrease between *Z. schneideriana* and *Phyllostachys edulis* (Carrière) J.Houz. Zhang et al. [21] found that total suitable area for *Betula albosinensis* Burkill could increase under some 2090s scenarios, while moderately and highly suitable areas contracted and highly suitable areas became more fragmented. Ni et al. [22] similarly reported greater fragmentation and lower connectivity despite increased vegetation area and cover in the Cangshan–Erhai National Nature Reserve. Area expansion therefore need not improve spatial structure, and resource assessments should also consider patch size, aggregation, isolation, and overlap among species.

Projections also depend on climate inputs and binarization methods. Stewart et al. [23] found substantial differences among plant distribution predictions based on different climate datasets and advocated ensembles that report spatial agreement and uncertainty. Rose et al. [24] compared the contributions of consensus methods, global climate models (GCMs), and emissions scenarios to predictive uncertainty, evaluating them separately across periods and dispersal assumptions. Threshold choice introduces further uncertainty: Hellegers et al. [25] found marked differences in predicted ranges among four methods and recommended selecting thresholds according to study objectives or comparing multiple methods. For presence-only data, some methods are sensitive to pseudo-absence specification, subjective choices, and validation data [26]. Threshold-dependent changes in binary suitable area can consequently affect ecological interpretation and management [27]. A common multi-GCM framework combined with threshold comparisons can help distinguish uncertainty arising from climate inputs and binarization.

Previous studies provide a foundation for understanding bamboo responses to climate change and uncertainty in distribution projections. However, differences in study systems, spatial scales, and model settings leave the relative responses of the three food bamboo species and their combined spatial patterns under common modeling conditions unresolved. Patch attributes can complement species distribution analyses [28]. A regional comparison should therefore consider suitable area, prediction uncertainty, patch structure, and spatial relationships among bamboo species together to inform conservation assessments of these plants and their potential role as food resources.

We selected these three taxa because they are documented giant panda food bamboos in this region, collectively represent the contrasting elevational positions recorded in the study area, and provide a common regional comparison; other food bamboos, including *Bashania fargesii* and *Fargesia* species, were outside the present dataset and study scope [5,10]. The species are presented in descending order of median elevation derived from the digital elevation model (DEM) in the final modeling records, consistent with the high-to-low elevational comparison (Table S14).

To address these gaps, we compared the potential distributions of and projected responses to climate change in *Bashania faberi*, *Yushania brevipaniculata*, and *Chimonobambusa szechuanensis* (Rendle) Keng f. within a defined study area in the Qionglai Mountains region of Sichuan, China using common predictor configurations, MaxEnt settings, and a multi-GCM ensemble projection framework. Specifically, we addressed the following questions: (1) Do the three food bamboo species differ in patterns of predictor contributions and marginal responses? (2) How do their potential suitable areas, spatial distributions, and inter-GCM disagreement change under different climate scenarios, and do conclusions about these changes depend on threshold choice and predictor combinations? (3) How do patch structure for individual species, the landscape configuration of their combined suitable areas, and spatial overlap among species change under future climate scenarios?

## 2. Results

### 2.1. Model Performance and Environmental Variable Contributions

Unless stated otherwise, suitability and landscape results refer to the nine-variable bootstrap ensemble, with future predictions averaged equally across three GCMs using the original 2.5-arc-minute future climate products interpolated to the 30-arc-second model grid. Predictor-configuration comparisons are presented in Section 2.5 and Tables S1–S5 and S13. Resolution, threshold and patch-connectivity diagnostics are reported in Section 2.4.3 and Section 2.4.4 and Tables S7 and S9; model-complexity, environmental-transfer, and GCM/bootstrap diagnostics are presented in Section 2.6, Section 2.7 and Section 2.8, Tables S6, S8 and S10, and Figures S1 and S2. Section 2.1 and Table S11 report spatial-background and environmental-coverage diagnostics, with validation partitions in Figure S3. Terrain-provenance checks and separate aspect and slope validation diagnostics are also reported in Section 2.1 and Table S15. Section 2.4.5 and Table S17 describe the separately distributed 30-arc-second future-product diagnostic, which does not replace the production baseline. Outcomes calculated from ensemble maps are distinguished from those calculated separately for each GCM.

Under the nine-variable baseline configuration, mean test AUC (area under the receiver operating characteristic curve) values for *Bashania faberi*, *Yushania brevipaniculata*, and *Chimonobambusa szechuanensis* in the original spatial four-fold validation with full-domain background were 0.9308, 0.8593, and 0.9083, respectively, with between-fold standard deviations of 0.0727, 0.1218, and 0.0614, respectively. Using the 10th percentile of predicted suitability at the training points in each fold as the threshold yielded mean test omission rates of 37.50%, 20.83%, and 37.05%, respectively (Table 1). The models showed some ability to discriminate presence points from background locations, but performance varied among spatial partitions, and omission rates were high in some validation folds. Mean AUC alone therefore does not establish reliable prediction across spatial partitions. Because each fold for *B. faberi* contained only 3–4 test points, its validation metrics and local prediction boundaries should be interpreted with caution.

Environmental-range diagnostics were extended to all three species and both predictor configurations (Table S11; Figure S3). In the nine-variable configuration, 14/15 *B. faberi*, 11/25 *Y. brevipaniculata*, and 19/43 *C. szechuanensis* test records lay outside at least one corresponding training-occurrence marginal range, using sine and cosine components for the aspect diagnostic. The respective counts outside the geographically partitioned training-background ranges were 3/15, 5/25, and 1/43. Environmental coverage inferred from training occurrences therefore differed from that inferred from the available background. These descriptive range checks do not establish the cause of omission or test novelty in multivariate combinations.

Across the final modeling records, DEM-derived elevations ranged from 1995 to 3215 m (median, 2811 m; *n* = 15) for *Bashania faberi*, from 1670 to 2587 m (median, 2136 m; *n* = 25) for *Yushania brevipaniculata*, and from 1265 to 2191 m (median, 1664 m; *n* = 43) for *Chimonobambusa szechuanensis* (Table S14). These raster-derived values describe the retained modeling records and should not be interpreted as field-measured elevations or full species elevational limits. Across the retained records, baseline mean diurnal temperature range (BIO2) was 10.33–11.99, 8.51–11.02, and 7.85–9.36 °C, and annual precipitation (BIO12) was 713–804, 756–1028, and 908–1167 mm for *B. faberi*, *Y. brevipaniculata*, and *C. szechuanensis*, respectively (Table S14). These climatic values were extracted from the 1970–2000 layers and do not represent weather measured during the 2025 surveys. Relative to initial records with complete values for all nine predictors, the final records retained 15 of 15, 25 of 33, and 43 of 52 occupied environmental cells for *B. faberi*, *Y. brevipaniculata*, and *C. szechuanensis*, respectively (Table S14). The maximum DEM-derived elevation for *Y. brevipaniculata* decreased from 2670 to 2587 m after processing; the maximum BIO12, BIO13, and BIO16 values for *C. szechuanensis* decreased by 3, 1, and 2 mm, respectively. The other minimum and maximum values among the eight non-circular predictors were unchanged.

Restricting evaluation to the aligned held-out background, without refitting the original nine-variable models, changed mean test AUC from 0.9308, 0.8593, and 0.9083 to 0.9526, 0.8791, and 0.8716 for *B. faberi*, *Y. brevipaniculata*, and *C. szechuanensis*, respectively; omission counts were unchanged. Diagnostic models refitted with geographically partitioned training backgrounds yielded mean AUC values of 0.9570, 0.8838, and 0.8591, and pooled omissions of 5/15 (33.33%), 5/25 (20.00%), and 12/43 (27.91%), respectively. Their five-variable counterparts omitted 4/15 (26.67%), 5/25 (20.00%), and 9/43 (20.93%). However, *B. faberi* fold 1 still omitted all four test records in both configurations, and *Y. brevipaniculata* fold 2 still omitted four of six. Thus, geographically partitioning the background did not eliminate fold-level prediction failures, and changes in AUC were not uniformly positive. These supplementary fits do not replace the production ensembles or the original validation values in Table 1 and Table S2.

Supplementary aspect diagnostics replaced angular aspect with cosine and sine components, either calculated from the existing model-grid values or interpolated from the archived fine-resolution aspect raster (Table S15). For *B. faberi*, *Y. brevipaniculata*, and *C. szechuanensis*, respectively, mean test AUCs under the nine-variable baseline were 0.9308, 0.8593, and 0.9083; they were 0.9328, 0.8689, and 0.9148 after model-grid conversion and 0.9328, 0.8631, and 0.9147 after fine-grid component interpolation. The component-based configurations contained ten rather than nine predictors. For *B. faberi*, pooled omissions remained 6/15 for all representations based on the nine-variable set and 4/15 for those based on the five-variable set. Thus, the aspect diagnostics did not resolve its high omission rates.

A numerical reconstruction using a constant horizontal conversion of 111,000 m per degree reproduced the archived model-grid slope with a mean absolute difference of 2.58 × 10^−6^° and a maximum of 1.16 × 10^−5^° across 7333 cells (Table S15). This numerical agreement does not establish the effective historical software settings. Using latitude-dependent WGS84 local distances instead increased slope by a mean of 1.554°, with a maximum absolute difference of 4.174°. Replacing only slope in the validation models changed mean test AUC by at most 0.0007 in absolute terms across the six species–configuration comparisons. Pooled omissions were unchanged in five comparisons; for *C. szechuanensis* with five predictors, they increased from 15/43 to 16/43. Unchanged pooled counts did not imply identical fold-level omissions.

Original full-domain-background fold-level results are provided in Table S2. Under the nine-variable baseline, *Bashania faberi* had 4/4, 2/4, 0/4, and 0/3 omitted test presences in folds 1–4, respectively (6/15 pooled; 40.00%), compared with an arithmetic mean omission rate of 37.50%. The corresponding pooled omission rates were 20.00% (5/25) for *Yushania brevipaniculata* and 37.21% (16/43) for *Chimonobambusa szechuanensis*; their arithmetic mean rates were 20.83% and 37.05%, respectively. Under the five-variable low-correlation configuration, pooled omission rates were 26.67% (4/15), 20.00% (5/25), and 34.88% (15/43), respectively (Table S2). Because all original folds used the same 7333 background cells, these values evaluate spatially withheld presences against a common full-domain background and do not constitute independent external validation.

**Figure 1:**
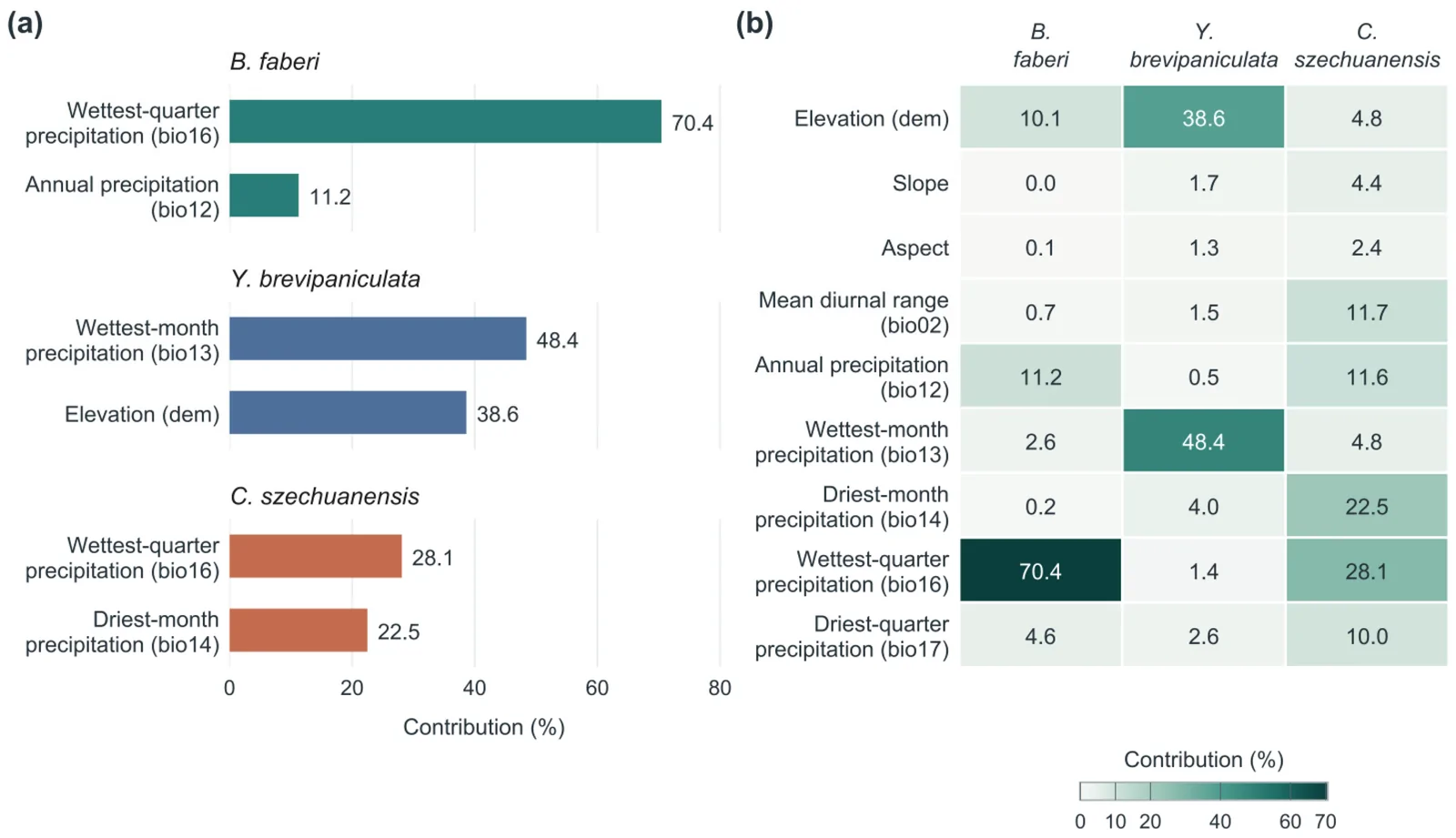
Percent contributions of environmental variables in the models for the three food bamboo species. (**a**) The two environmental variables with the highest percent contributions in the models for *Bashania faberi*, *Yushania brevipaniculata*, and *Chimonobambusa szechuanensis*; (**b**) matrix of percent contributions of the nine environmental variables across the three species models. Values at the ends of bars, values within matrix cells, and the color scale all indicate variable contributions (%).

Predictor contributions differed markedly among the three food bamboo species under the predictor set and model settings used here. In the *Bashania faberi* model, precipitation of the wettest quarter (bio16, 70.43%) made the largest contribution, followed by annual precipitation (bio12, 11.24%) and elevation (dem, 10.06%). In the *Yushania brevipaniculata* model, precipitation of the wettest month (bio13, 48.39%) and elevation (dem, 38.61%) contributed substantially. In the *Chimonobambusa szechuanensis* model, precipitation of the wettest quarter (bio16, 28.08%) and precipitation of the driest month (bio14, 22.47%) were the major contributors. Overall, elevation and precipitation variables made relatively large contributions in all three models (Figure 1).

**Figure 2:**
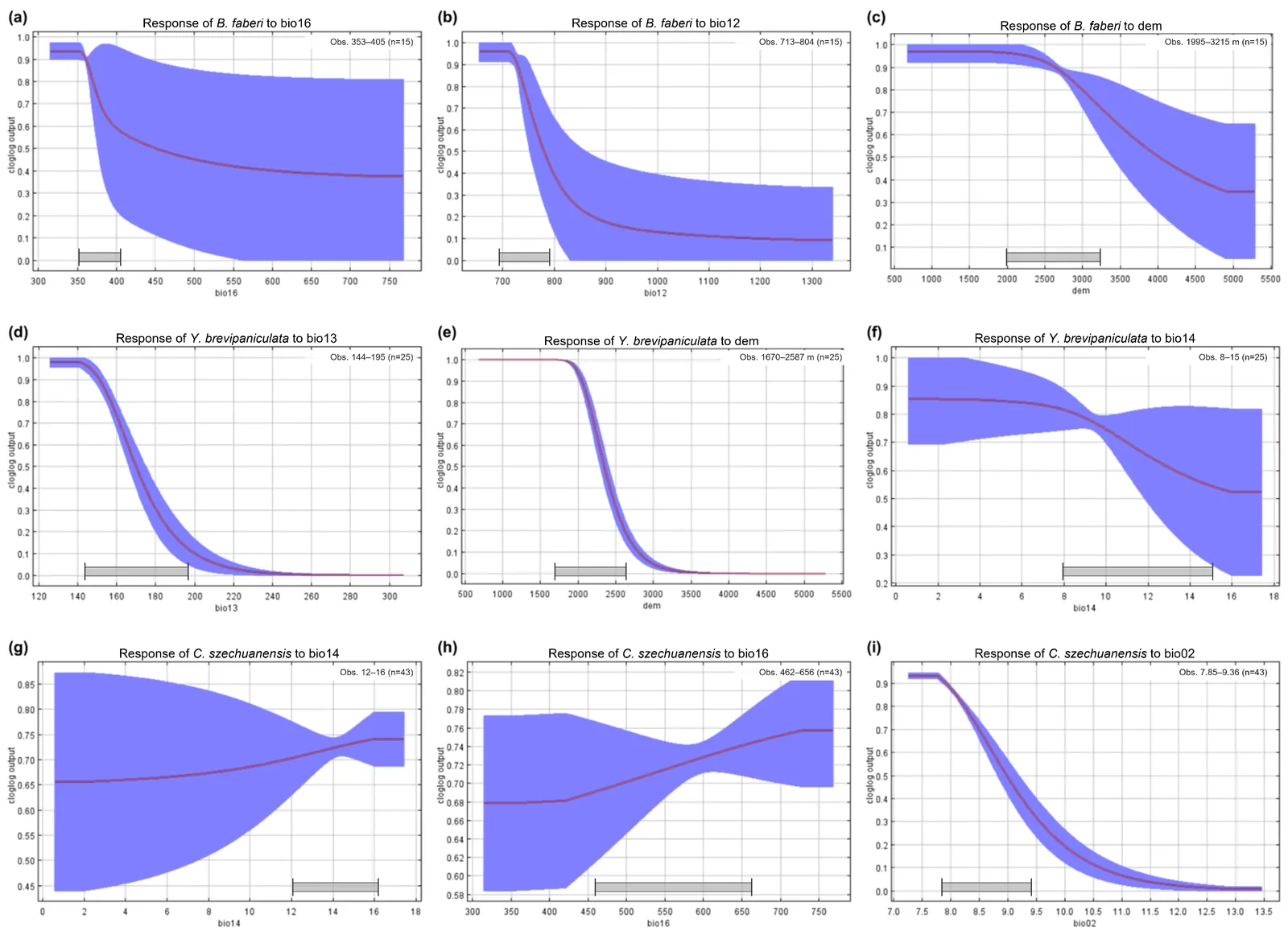
Marginal response curves for the main environmental variables of the three food bamboo species. (**a**–**c**) Responses of *Bashania faberi* to precipitation of the wettest quarter (bio16), annual precipitation (bio12), and elevation (dem); (**d**–**f**) responses of *Yushania brevipaniculata* to precipitation of the wettest month (bio13), elevation (dem), and precipitation of the driest month (bio14); (**g**–**i**) responses of *Chimonobambusa szechuanensis* to precipitation of the driest month (bio14), precipitation of the wettest quarter (bio16), and mean diurnal temperature range (bio02). Red lines indicate mean responses across 10 bootstrap replicates and blue shading represents ±1 standard deviation around the mean. All other environmental variables were held at their mean values in the training samples. The horizontal axes show precipitation in millimeters, elevation in meters, and mean diurnal temperature range in degrees Celsius. The vertical axes indicate complementary log–log (cloglog) predicted suitability. Marginal predictor ranges extracted at the final occurrence records are summarized in Supplementary Table S14; these raster-extracted values do not represent independent field measurements. In each panel, the gray range strip and end markers show the minimum-to-maximum value of the corresponding predictor extracted from the final modeling records; these marks are distinct from the blue ±1 SD response band and do not represent the full niche limits. Figure S5 compares the elevation responses with predictions under actual environmental combinations and documents their environmental support.

Marginal response curves also differed among the three food bamboo species. For *B. faberi*, predicted suitability generally decreased with increasing precipitation of the wettest quarter, annual precipitation, and elevation. For *Y. brevipaniculata*, predicted suitability decreased with wettest-month precipitation and elevation; it declined at higher driest-month precipitation values. For *C. szechuanensis*, predicted suitability declined with mean diurnal temperature range. Responses to wettest-quarter and driest-month precipitation were relatively flat, with a slight increase at higher wettest-quarter precipitation values. The field records and current predictions indicate different elevation distributions with some local overlap. Percent contribution represents the relative contribution of each variable during model fitting, whereas marginal response curves show how predicted suitability changes with one variable while all others are held at their mean values in the training samples. Neither provides direct evidence of independent ecological effects or definitive ecological suitability limits (Figure 2). The DEM ranges describe the retained records, not complete ecological limits, and conditional response curves need not reproduce observed elevation frequencies.

To examine this distinction directly, we inspected the archived fitted models and evaluated their predictions over actual environmental combinations (Figure S5). Nine of the ten *B. faberi* replicates and all ten *Y. brevipaniculata* replicates contained negative elevation-related product or quadratic terms; the remaining *B. faberi* replicate had no active elevation term. At 1000–<1500 m, the *B. faberi* curves held annual precipitation at training means of 723.93–745.33 mm, below the 963–1282 mm range present in grid cells at those elevations. The corresponding *Y. brevipaniculata* training means for wettest-month precipitation were 155.36–161.60 mm, compared with 186–292 mm in those cells. Thus, the low-elevation portions of the fixed-mean curves include environmental combinations not represented in that elevation band. With actual joint environmental values, mean predictions across 500 m bands peaked at 3000–<3500 m for *B. faberi* and 2000–<2500 m for *Y. brevipaniculata*. These descriptive peaks depend on band width and are not estimated ecological optima. The diagnostic demonstrates how model conditioning and available environmental combinations produce different elevation summaries; it does not establish an independent effect of elevation or provide new field validation.

### 2.2. Current Potential Distribution Patterns and Interspecific Differences

**Figure 3:**
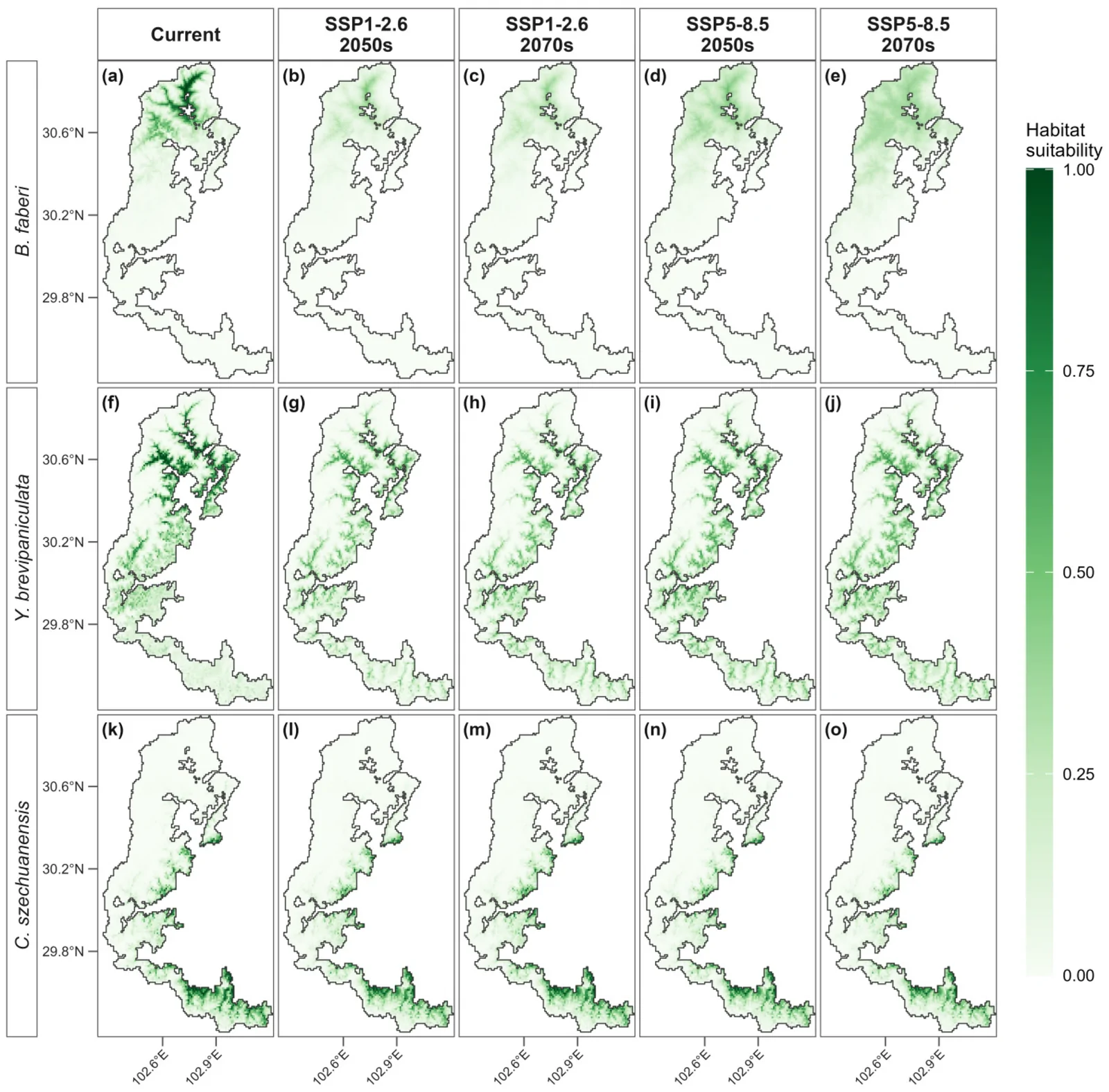
Mean suitability under current climatic conditions and three-GCM ensemble-mean suitability under future climate scenarios for the three food bamboo species. (**a**–**e**) *Bashania faberi*; (**f**–**j**) *Yushania brevipaniculata*; (**k**–**o**) *Chimonobambusa szechuanensis*. Columns from left to right represent current conditions, SSP1-2.6 in the 2050s, SSP1-2.6 in the 2070s, SSP5-8.5 in the 2050s, and SSP5-8.5 in the 2070s. SSP denotes Shared Socioeconomic Pathway. Future predictions are pixel-wise means of suitability projections from three global climate models: BCC-CSM2-MR, MIROC6, and MPI-ESM1-2-HR. The color scale indicates cloglog predicted suitability. The 2050s and 2070s represent 2041–2060 and 2061–2080, respectively.

Under the 1970–2000 climatic baseline, the three food bamboo species had different predicted patterns of potential suitability. Using a fixed threshold of 0.6, the areas with complementary log–log (cloglog) predicted values ≥ 0.6 for *Bashania faberi*, *Yushania brevipaniculata*, and *Chimonobambusa szechuanensis* were 164.38, 389.36, and 255.44 km^2^, respectively. The continuous suitability maps showed that areas with relatively high predicted suitability for *B. faberi* were concentrated mainly at higher elevations in the northern part of the study area; the predicted suitable area for *Y. brevipaniculata* was more extensive, occurring primarily in the central and north-central parts of the study area; and areas with relatively high predicted suitability for *C. szechuanensis* were located mainly at lower elevations in the southern and southeastern parts of the study area. The predicted distributions were spatially differentiated, with some local overlap. The first column of Figure 3 presents the current continuous suitability for the three species. Species-specific 10th-percentile presence (10P) thresholds were used for the suitable-area analysis in Section 2.3.1 and the baseline landscape analyses in Section 2.4.1, Section 2.4.2 and Section 2.4.3. The fixed threshold of 0.6 characterizes areas with relatively high predicted values; suitable-area comparisons among all three thresholds are presented in Section 2.3.2, and landscape threshold sensitivity is reported in Section 2.4.4.

### 2.3. Suitable-Area Dynamics and Uncertainty Under Future Climate Change

#### 2.3.1. Suitable-Area Dynamics Under Future Climate Change

Based on ensemble-mean projections from three GCMs (BCC-CSM2-MR, MIROC6, and MPI-ESM1-2-HR), projected suitable areas varied among species and scenarios. Using species-specific 10P thresholds, the suitable areas of *Bashania faberi* and *Yushania brevipaniculata* were below current levels under Shared Socioeconomic Pathway (SSP) scenario SSP1-2.6 in both the 2050s and 2070s and under SSP5-8.5 in the 2050s, but exceeded current levels under SSP5-8.5 in the 2070s. The suitable area of *Chimonobambusa szechuanensis* was below the current level under all four future scenarios (Figure 3; Table 2).

At the 10P thresholds, current suitable areas were 435.83 km^2^ for *B. faberi* and 1052.92 km^2^ for *Y. brevipaniculata*. Their future areas ranged from 59.71 to 472.72 km^2^ and from 706.45 to 1119.12 km^2^, respectively. Both reached their largest projected areas under SSP5-8.5 in the 2070s (Table 2).

For *C. szechuanensis*, suitable area declined from 607.55 km^2^ to 434.08–558.37 km^2^, with the smallest area under SSP5-8.5 in the 2070s. Figure 3 shows continuous cloglog suitability, whereas Table 2 reports areas meeting the species-specific 10P thresholds.

**Figure 4:**
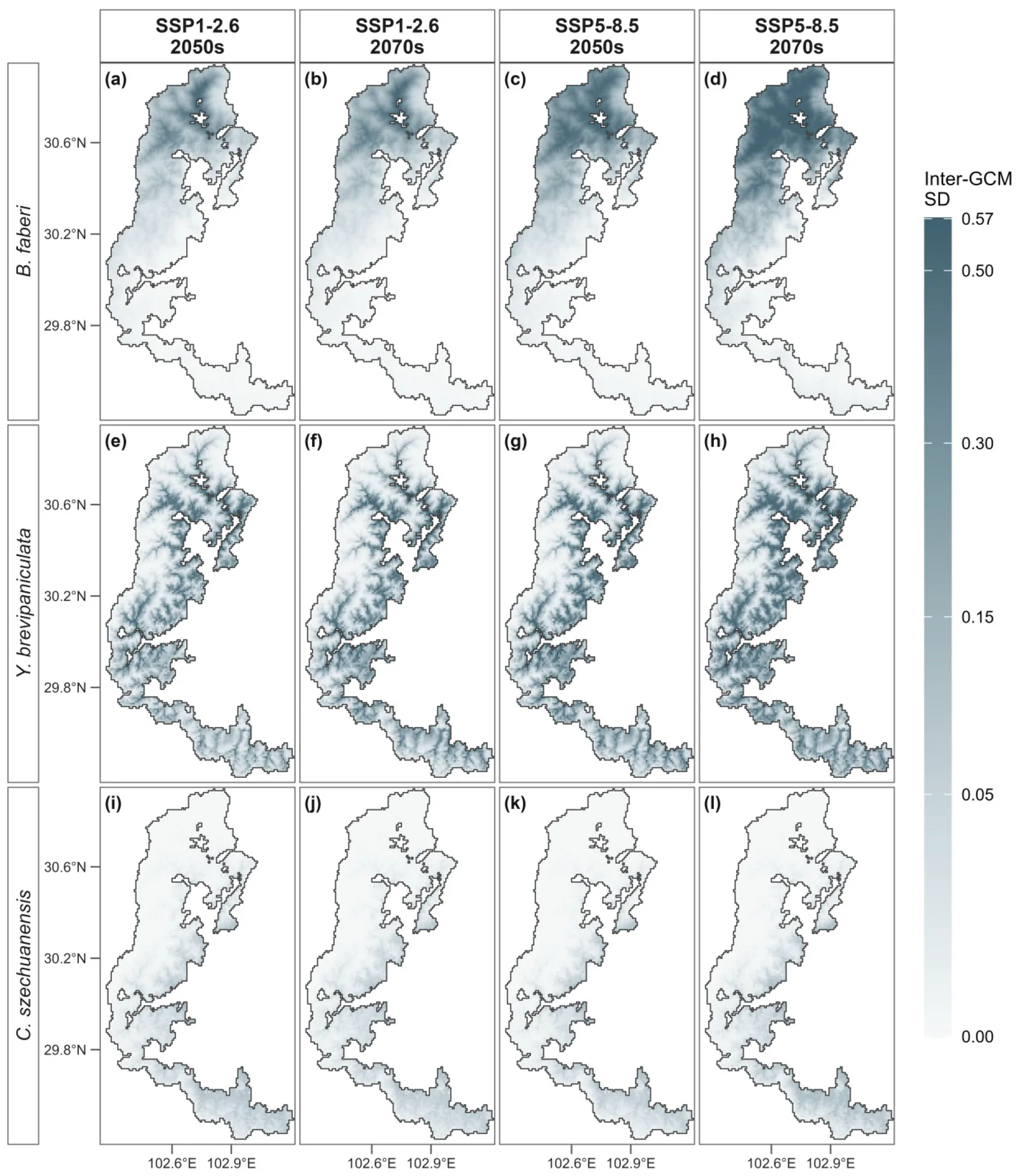
Spatial distributions of standard deviations among global climate models (GCMs) in future suitability projections for the three food bamboo species. (**a**–**d**) *Bashania faberi*; (**e**–**h**) *Yushania brevipaniculata*; (**i**–**l**) *Chimonobambusa szechuanensis*. Columns from left to right represent SSP1-2.6 in the 2050s, SSP1-2.6 in the 2070s, SSP5-8.5 in the 2050s, and SSP5-8.5 in the 2070s. SSP denotes Shared Socioeconomic Pathway. For each GCM, the pixel-wise mean of 10 replicate projections was first calculated. Pixel-wise sample standard deviations were then calculated across the mean predictions from BCC-CSM2-MR, MIROC6, and MPI-ESM1-2-HR using a denominator of 3 − 1. Colors from light blue-gray to dark blue-gray indicate increasing disagreement among projections. The color scale uses a square-root transformation, while legend ticks display untransformed standard deviation values. The 2050s and 2070s represent 2041–2060 and 2061–2080, respectively.

Inter-GCM disagreement differed among species. Across the four future scenarios, spatial mean standard deviations ranged from 0.0552 to 0.1603 for *Bashania faberi*, from 0.1029 to 0.1661 for *Yushania brevipaniculata*, and from 0.0098 to 0.0146 for *Chimonobambusa szechuanensis*. *Yushania brevipaniculata* had the highest value in every scenario, followed by *B. faberi* and *C. szechuanensis*. For the first two species, disagreement peaked under SSP5-8.5 in the 2070s (Figure 4). These means summarize pixel-level standard deviations across the jointly valid study domain and quantify disagreement among the three selected GCMs, not total uncertainty or independently validated accuracy.

#### 2.3.2. Threshold Sensitivity Analysis

Threshold choice affected both the extent of suitable areas and, for some species and scenarios, the direction of projected area change. For *Bashania faberi*, future suitable areas under the 10P threshold ranged from 59.71 to 472.72 km^2^, exceeding the current area under SSP5-8.5 in the 2070s. Under the fixed threshold of 0.5, the areas in the four future scenarios were 0.74, 0, 2.95, and 0 km^2^, respectively, all below the current area of 232.25 km^2^. Under the fixed threshold of 0.6, suitable areas were 0 km^2^ in all four future scenarios, compared with the current area of 164.38 km^2^. Thus, the increase in suitable area for *B. faberi* under the 10P threshold for SSP5-8.5 in the 2070s was not accompanied by an increase in the area with predicted values ≥0.5 or ≥0.6.

For *Yushania brevipaniculata*, suitable area under the 10P threshold likewise exceeded the current area under SSP5-8.5 in the 2070s but remained below it under the other scenarios. Under the fixed threshold of 0.5, future suitable areas ranged from 218.31 to 356.96 km^2^, all below the current area of 531.38 km^2^. Under the fixed threshold of 0.6, future suitable areas ranged from 71.73 to 139.11 km^2^, all below the current area of 389.36 km^2^. For *Chimonobambusa szechuanensis*, suitable areas in all four future scenarios were smaller than the corresponding current areas under all three thresholds. Future suitable areas under the fixed thresholds of 0.5 and 0.6 ranged from 204.77 to 299.36 km^2^ and from 133.29 to 206.29 km^2^, respectively (Table 3).

Within the nine-variable baseline ensemble, the projected decline in suitable area for *C. szechuanensis* persisted across the three thresholds, whereas the direction of suitable-area change for *B. faberi* and *Y. brevipaniculata* under SSP5-8.5 in the 2070s depended on threshold selection. An increase in area at one threshold therefore does not establish an overall improvement in suitability. Conclusions about species responses were not uniformly supported across all three thresholds.

### 2.4. Landscape Changes Across Scales and Spatial Relationships Among Bamboo Species

#### 2.4.1. Landscape Changes in Suitable Areas of Individual Species

**Figure 5:**
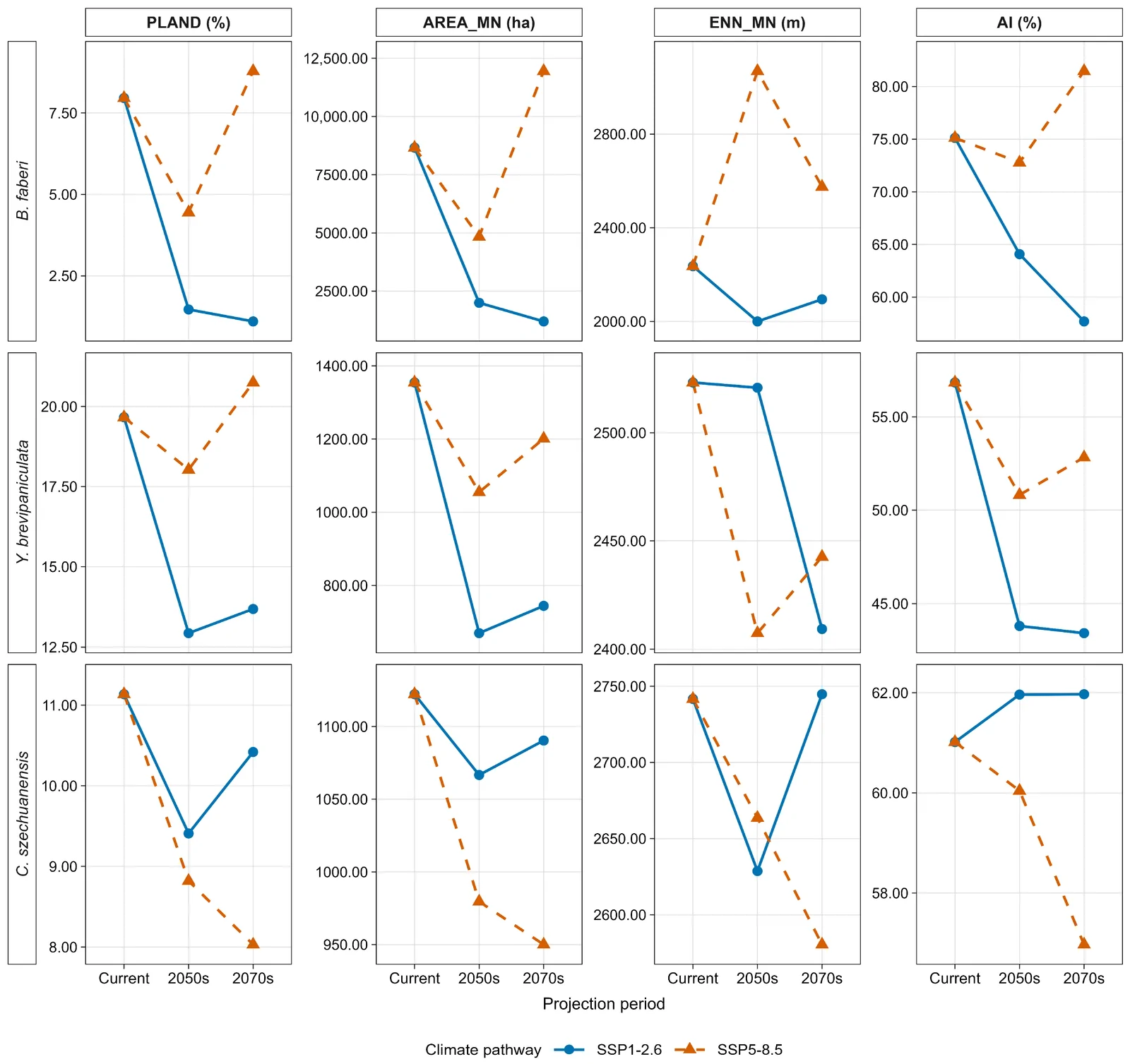
Changes in landscape metrics for suitable areas of the three food bamboo species based on the 10P threshold. Rows represent *Bashania faberi*, *Yushania brevipaniculata*, and *Chimonobambusa szechuanensis*, respectively. Columns represent percentage of landscape (PLAND, %), mean patch area (AREA_MN, ha), mean Euclidean nearest-neighbor distance (ENN_MN, m), and aggregation index (AI, %), respectively. Blue solid lines with circles indicate SSP1-2.6 and orange dashed lines with triangles indicate SSP5-8.5; both trajectories share the same current climatic baseline value. Vertical-axis ranges are set independently for each panel. 10P denotes the 10th-percentile presence threshold and SSP denotes Shared Socioeconomic Pathway. Landscape metrics were calculated on a 1 km equal-area grid with eight-neighbor patch delineation; AI used shared-edge like adjacencies.

Landscape metrics derived from binary suitability maps at the 10P threshold showed that projected landscape changes varied among species and scenarios (Figure 5; Table 4). For *Bashania faberi*, current values of percentage of landscape (PLAND), mean patch area (AREA_MN), and aggregation index (AI) were 7.96%, 8660.00 ha, and 75.12%, respectively. Under SSP1-2.6 in the 2050s and 2070s and SSP5-8.5 in the 2050s, PLAND ranged from 1.10% to 4.45%, AREA_MN from 1200.00 to 4840.00 ha, and AI from 57.69% to 72.79%, all below their current values. Under SSP5-8.5 in the 2070s, these metrics increased to 8.78%, 11,950.00 ha, and 81.47%, respectively, while the largest patch index (LPI) also increased from 5.66% to 8.62%. The future number of patches (NP) ranged from 4 to 5; mean Euclidean nearest-neighbor distance (ENN_MN) ranged from 2000.00 to 3069.48 m, with the direction of change varying among scenarios. Thus, future landscape changes for *B. faberi* did not consistently indicate a loss of suitable area or a decline in large-patch structure across scenarios.

For *Yushania brevipaniculata*, current PLAND was 19.66%, compared with 12.94%, 13.69%, 18.03%, and 20.75% under the four future scenarios, respectively; only the value under SSP5-8.5 in the 2070s exceeded the current value. AREA_MN decreased from 1354.43 ha to 670.48–1201.06 ha, AI declined from 56.85% to 43.41–52.83%, and NP increased from 79 to 93–105. Future ENN_MN ranged from 2407.41 to 2520.81 m, below the current value of 2523.23 m in all scenarios, although the difference under SSP1-2.6 in the 2050s was minimal. These results indicate that an increase in suitable area for *Y. brevipaniculata* under some scenarios does not necessarily coincide with increases in mean patch size or aggregation. Patch number, mean patch area, aggregation, and nearest-neighbor distance did not all change in the same direction.

For *Chimonobambusa szechuanensis*, PLAND decreased from 11.14% to 8.03–10.42%, AREA_MN declined from 1122.22 ha to 950.00–1090.38 ha, and NP decreased from 54 to 46–52. Under SSP1-2.6, AI was 61.96% in the 2050s and 61.97% in the 2070s, slightly above the current value of 61.02%; under SSP5-8.5, it declined to 60.04% and 56.97%, respectively. Future ENN_MN ranged from 2580.41 to 2744.77 m, slightly exceeding the current value of 2741.69 m under SSP1-2.6 in the 2070s but remaining below it under the other scenarios. Thus, both the proportion of the landscape occupied by suitable areas and mean patch area declined for *C. szechuanensis*, whereas changes in aggregation and interpatch distance varied among scenarios.

The landscape metrics therefore indicate species- and scenario-specific responses rather than uniform deterioration. They do not, by themselves, support a single overall risk ranking for the three species.

#### 2.4.2. Combined Suitable-Area Patterns and Spatial Relationships Among Species

**Figure 6:**
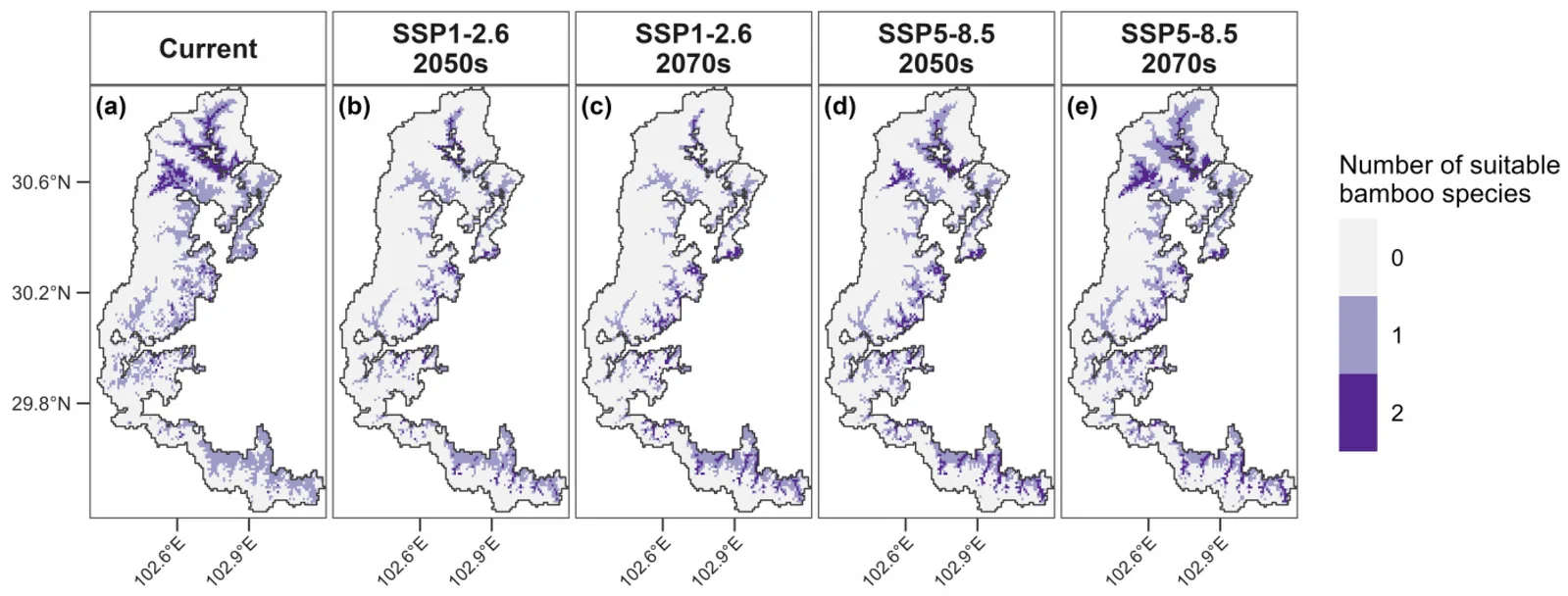
Spatial patterns of suitable bamboo species richness under current and future climatic conditions based on the 10P threshold. (**a**) Current conditions; (**b**) SSP1-2.6 in the 2050s; (**c**) SSP1-2.6 in the 2070s; (**d**) SSP5-8.5 in the 2050s; (**e**) SSP5-8.5 in the 2070s. Pixel values indicate the number of food bamboo species meeting their respective 10P thresholds within each pixel. The theoretical range is 0–3, whereas the values occurring under all five climatic conditions shown range from 0 to 2. The 2050s and 2070s represent 2041–2060 and 2061–2080, respectively.

Summing the binary suitability maps at the species-specific 10P thresholds produced a map of the number of bamboo species for which each pixel was predicted to be suitable (hereafter, suitable bamboo species richness) (Figure 6). Although pixel values could theoretically range from 0 to 3, the predicted values ranged from 0 to 2 under both current conditions and all four future climate scenarios. No pixels met the species-specific 10P thresholds for all three species simultaneously. This pattern describes overlap among predicted suitable areas under the specified models and thresholds; it does not establish coexistence in the field.

**Figure 7:**
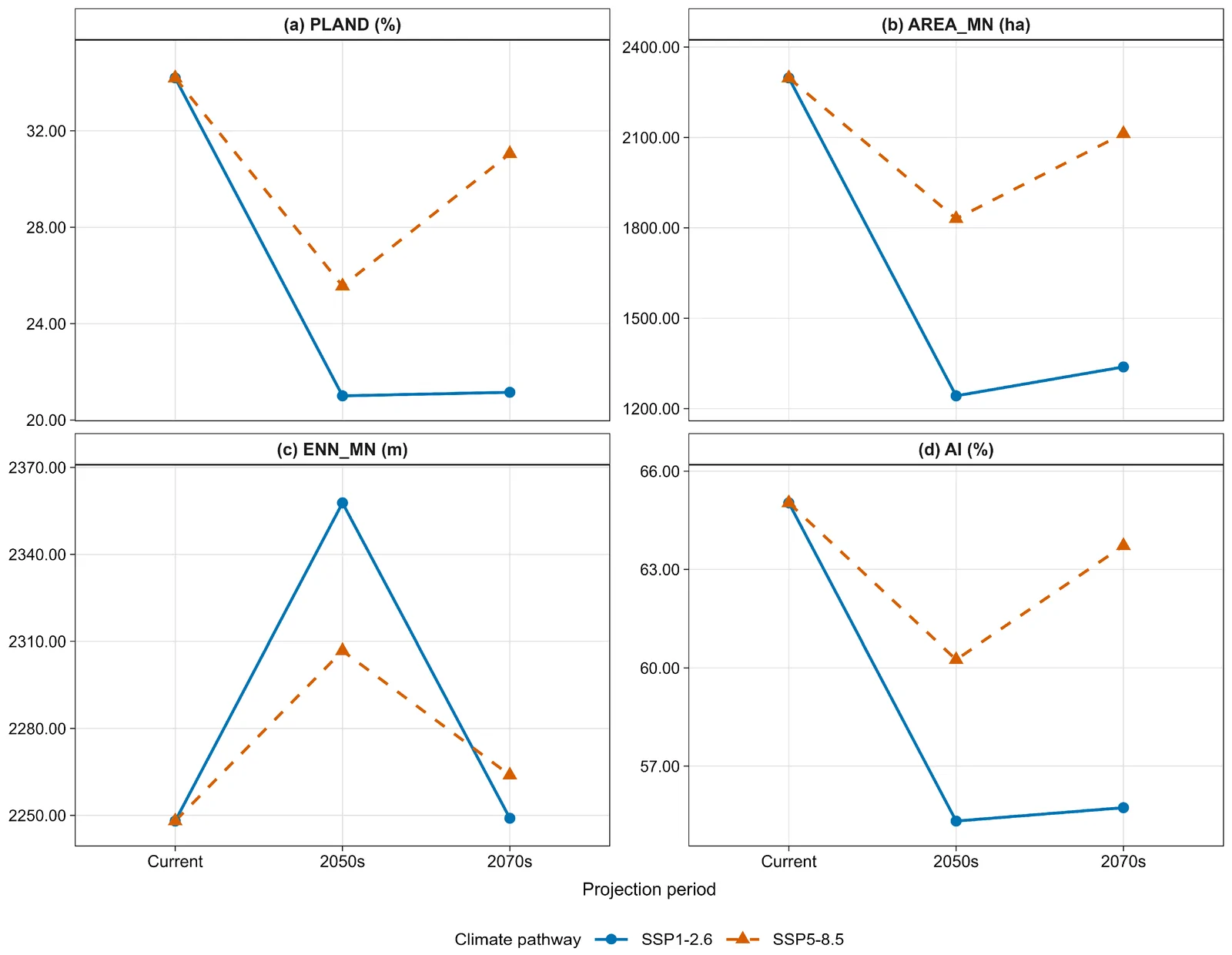
Changes in landscape metrics for the combined suitable area of the three food bamboo species based on the 10P threshold. (**a**) Percentage of landscape (PLAND, %); (**b**) mean patch area (AREA_MN, ha); (**c**) mean Euclidean nearest-neighbor distance (ENN_MN, m); (**d**) aggregation index (AI, %). The combined suitable area comprises pixels where at least one food bamboo species meets its species-specific 10P threshold. Blue solid lines with circles indicate SSP1-2.6 and orange dashed lines with triangles indicate SSP5-8.5; both trajectories share the same current climatic baseline value. Landscape metrics were calculated on a 1 km equal-area grid with eight-neighbor patch delineation; AI used shared-edge like adjacencies.

The combined suitable area, used here to represent potential bamboo resources, was defined as the union of pixels where at least one species met its species-specific 10P threshold. Its landscape metrics showed that PLAND decreased from 34.20% under current conditions to 21.00–31.05% in the future, AREA_MN declined from 2297.53 ha to 1242.39–2112.50 ha, and AI decreased from 65.03% to 55.32–63.72%. Current NP was 81, compared with 92, 86, 76, and 80 under SSP1-2.6 in the 2050s, SSP1-2.6 in the 2070s, SSP5-8.5 in the 2050s, and SSP5-8.5 in the 2070s, respectively, indicating an increase under SSP1-2.6 and a decrease under SSP5-8.5. The landscape proportion, mean patch area, and aggregation of the combined suitable area were lower than their current values under all four future scenarios, whereas patch number did not consistently increase (Figure 7; Table 5).

ENN_MN for the combined suitable area increased from 2248.03 m to 2249.01–2357.78 m, although the increase under SSP1-2.6 in the 2070s was only approximately 1 m. Under SSP5-8.5 in the 2070s, LPI rose from 6.17% to 10.11%, while PLAND and AREA_MN remained below current levels at 31.05% and 2112.50 ha. These contrasting changes show why an increase in one metric cannot establish overall improvement in spatial structure.

Schoener’s *D* indicated species-pair-specific changes in overlap between continuous suitability surfaces (Table 6). For *Bashania faberi* and *Yushania brevipaniculata*, D changed from 0.3027 to 0.2387–0.3105, exceeding the current value only under SSP5-8.5 in the 2070s. For *B. faberi* and *Chimonobambusa szechuanensis*, D increased from 0.0273 to 0.0287–0.0383 but remained low. For *Y. brevipaniculata* and *C. szechuanensis*, it increased from 0.3288 to 0.4146–0.4830 under all four future scenarios.

Taken together, the combined-area and overlap results show that changes in potential resources require joint consideration of suitable area, patch configuration, and interspecific spatial relationships.

#### 2.4.3. Resolution Sensitivity of Landscape Metrics

To assess sensitivity to the spatial grain of the prediction surfaces, continuous predictions were aggregated to a common 2.5 arc-minute grid and landscape metrics were calculated on a 5 km equal-area grid, using a mask derived directly from the original valid-data domain. Under the nine-variable configuration, 10P thresholds, and eight-neighbor patch delineation, combined PLAND declined from 33.33% to 20.00–30.67%, AREA_MN from 17,045.45 to 7031.25–15,681.82 ha, LPI from 16.89% to 5.78–11.56%, and AI from 50.00% to 31.58–45.45%. NP changed from 11 to 11–16 and ENN_MN increased from 10,753.12 m to 10,821.63–11,466.71 m. For *B. faberi*, future PLAND ranged from 0.44% to 7.56%, compared with 7.56% currently, and AREA_MN ranged from 2500.00 to 42,500.00 ha, compared with 42,500.00 ha currently. Some *B. faberi* metrics were undefined where only one patch or one suitable cell remained. Combined LPI declined in all four coarse-grid scenarios, whereas it increased under SSP5-8.5 in the 2070s on the 1 km grid. NP responses also depended on grain, while combined ENN_MN increased at both grains (Table S7).

#### 2.4.4. Threshold and Patch-Connectivity Sensitivity

The factorial sensitivity analysis comprised 480 combinations of two predictor configurations, two spatial grains, three thresholds, two patch-delineation rules, four resource layers, and five climatic conditions (Table S9). For the ensemble-mean predictions on the 1 km grid, combined PLAND and AI declined in all four future scenarios under both predictor configurations and all three thresholds; AREA_MN also declined under both four- and eight-neighbor patch definitions. Absolute patch summaries nevertheless depended strongly on the delineation rule. For example, under the current nine-variable 10P configuration, four-neighbor delineation produced 185 patches with an AREA_MN of 1005.95 ha, compared with 81 patches and 2297.53 ha under eight-neighbor delineation. PLAND was identical because the suitable-cell count was unchanged. AI was also identical because its shared-edge adjacency definition does not use the selected patch-delineation rule; this equality is not an independent test of connectivity robustness.

For the ensemble-mean predictions at the 5 km grain, combined PLAND, AREA_MN, and AI declined at the 10P thresholds under both configurations and patch definitions. Combined PLAND also declined at the fixed thresholds, but AREA_MN and AI did not decline uniformly. Under the nine-variable configuration, a threshold of 0.5, and four-neighbor delineation, AREA_MN increased from 4166.67 to 4583.33 ha under SSP1-2.6 in the 2070s; AI increased from 26.53% to 33.33% under the same configuration and scenario. At a threshold of 0.6, nine-variable AI increased from 27.59% to 40.00% under SSP1-2.6 in both periods. No combined suitable cells remained at the 5 km grain and a threshold of 0.6 under the nine-variable SSP5-8.5 2070s projection or any of the four five-variable future projections, leaving AREA_MN, ENN_MN, and AI undefined. These outcomes describe thresholded predictions at a coarse grain and do not demonstrate species extinction.

#### 2.4.5. Future Climate Product Sensitivity

A paired future climate product comparison retained the fitted models and replaced the interpolated 2.5-arc-minute inputs with the separately distributed WorldClim 30-arc-second products (Table S17). At 10P, the nine-variable ensemble projected future suitable areas of 123.09–538.40 km^2^ for *B. faberi*, 585.69–1054.40 km^2^ for *Y. brevipaniculata*, and 454.90–588.11 km^2^ for *C. szechuanensis*. The change directions in all 36 species-level ensemble comparisons at 10P were retained across the three predictor configurations and four future combinations. However, the nine-variable *Y. brevipaniculata* increase under SSP5-8.5 in the 2070s decreased from 66.20 to 1.48 km^2^. At a fixed threshold of 0.6, the BIO1-containing configuration changed from contraction to expansion for *Y. brevipaniculata* and the combined resource under SSP1-2.6 in both periods. Individual GCM outcomes also included changes in direction.

The future climate product also affected landscape summaries. With the official 30-arc-second inputs, the nine-variable 10P ensemble had combined PLAND of 21.70–33.66%, AREA_MN of 1283.70–2093.15 ha, AI of 56.34–65.06, and NP of 73–93. Under SSP5-8.5 in the 2070s, AI changed from a decrease of 1.31 to an increase of 0.03 points relative to the same current baseline, and NP changed from 80 under the original product to 92 under the alternative product, compared with 81 currently. This small AI increase is a numerical sensitivity, not evidence of ecologically meaningful improvement. The paired results show that stable species-level area directions at 10P do not imply stable patch counts, aggregation, or higher-threshold outcomes.

### 2.5. Sensitivity to Predictor Combinations

Under the five-variable low-correlation configuration, mean test AUCs from spatial four-fold cross-validation were 0.9362, 0.8847, and 0.9219 for *Bashania faberi*, *Yushania brevipaniculata*, and *Chimonobambusa szechuanensis*, respectively, with between-fold standard deviations of 0.0753, 0.0950, and 0.0370. Mean test omission rates were 25.00%, 20.83%, and 34.77%, respectively (Table S2). Although mean AUCs were higher than those under the nine-variable baseline configuration, omission rates remained high in some validation folds, and differences between configurations were not tested for statistical significance. These results therefore do not establish the five-variable configuration as the optimal model.

For the ensemble-mean predictions at the 10P threshold, the direction of suitable-area change was consistent between the original nine-variable and DEM-containing five-variable configurations for 10 of the 12 species–scenario combinations. Under SSP5-8.5 in the 2070s, the nine-variable baseline configuration projected increases in suitable area of 8.46% for *B. faberi* and 6.29% for *Y. brevipaniculata* relative to their current areas, whereas the five-variable configuration projected decreases of 17.65% and 3.87%, respectively. Under both fixed thresholds of 0.5 and 0.6, the direction of suitable-area change was consistent between the original two production configurations for all 12 species–scenario combinations. *Chimonobambusa szechuanensis* showed decreases in suitable area under the original two production configurations, all three thresholds, and all four future scenarios (Table S3). PLAND, AREA_MN, and AI for the combined suitable area were below their respective current values under all four future scenarios in the original two production configurations (Table S4), whereas the direction of change in Schoener’s *D* for some species pairs differed between variable configurations (Table S5).

The temperature-inclusive diagnostic produced a different response pattern. At its configuration-specific 10P threshold, ensemble suitable area for *Yushania brevipaniculata* increased from 1230.48 km^2^ under current conditions to 1699.06, 1710.31, 2331.19, and 3646.87 km^2^ across SSP1-2.6 in the 2050s and 2070s and SSP5-8.5 in the 2050s and 2070s, respectively. *Chimonobambusa szechuanensis* increased from 557.84 to 679.25, 691.18, 832.56, and 973.24 km^2^ and the combined suitable area increased from 2119.77 to 2344.28, 2204.51, 2784.82, and 4282.81 km^2^. *Bashania faberi* declined from 638.63 to 165.86, 45.70, and 126.79 km^2^ in the first three combinations but increased to 1138.58 km^2^ under SSP5-8.5 in the 2070s. Individual GCM directions were not unanimous: *B. faberi* had 1/3, 0/3, 0/3, and 1/3 increases; *Y. brevipaniculata* and the union had 2/3 increases in every combination; and *C. szechuanensis* had 2/3, 3/3, 3/3, and 3/3 increases. For the union, PLAND increased from 39.36% to 40.87–78.78%, AREA_MN from 3693 to 8237.04–61,242.86 ha, and AI from 69.70% to 79.14–91.25%. These results are conditional on replacing dem with BIO1 and therefore represent predictor-configuration sensitivity, not a temperature-only effect or unanimous GCM support (Supplementary Table S13).

### 2.6. Model-Complexity Sensitivity

Across the six configurations, mean test AUC ranged from 0.9303 to 0.9362 for *Bashania faberi*, 0.8440 to 0.8847 for *Yushania brevipaniculata*, and 0.9053 to 0.9218 for *Chimonobambusa szechuanensis*. Pooled test omission ranged from 26.67% to 40.00%, 16.00% to 24.00%, and 32.56% to 39.53%, respectively. The number of nonzero lambda terms also varied among feature and regularization settings (Table S6). In the full-data transfer diagnostic, future suitable-area direction across the 12 GCM-by-scenario–period combinations was mixed for every species and configuration. For the baseline LQP RM = 2 configuration, the numbers of decreases and increases were 8 and 4 for *B. faberi*, 7 and 5 for *Y. brevipaniculata*, and 10 and 2 for *C. szechuanensis*. These comparisons indicate that similar cross-validation scores do not guarantee stable future transfer; the alternative settings were therefore retained as sensitivity diagnostics rather than used to replace the production model.

### 2.7. Environmental Novelty and Clamping Sensitivity

Across the 12 GCM–scenario–period combinations, the proportion of the 7333 valid cells with negative multivariate environmental similarity surface (MESS) scores ranged from 0.08% to 41.21% under the nine-variable configuration and from 0% to 17.76% under the five-variable configuration. The corresponding area-weighted proportions were 0.08–41.36% and 0–17.83%, respectively. BCC-CSM2-MR produced the largest proportion of strict extrapolation in each scenario under both configurations. These diagnostics describe the environmental domain and are therefore shared among species within a predictor configuration (Table S8).

In the nine-variable, 10P production ensemble under SSP5-8.5 in the 2070s, newly suitable areas totaled 89.26 km^2^ for *B. faberi* and 388.23 km^2^ for *Y. brevipaniculata*. Of these gains, 66.11% and 96.94%, respectively, occurred in cells outside the calibration range for at least one GCM. No gains for these two species occurred in cells classified as strict extrapolation by all three GCMs. These gains represent transitions from unsuitable to suitable cells and differ from net area change after losses are subtracted. At the 10P thresholds, across future scenarios, 98.69–100% of the nine-variable ensemble’s suitable area for *C. szechuanensis* overlapped strict extrapolation in at least one GCM, compared with 38.19–92.28% under the five-variable configuration. Such overlap identifies a contributor to uncertainty; it does not mean that all three GCM projections extrapolate at those locations (Table S8).

In the full-data clamping comparison, mean absolute prediction differences across the domain reached 0.00393, and the largest local difference was 0.19757 on the cloglog scale. The proportion of cells with absolute differences >0.001 ranged from 0% to 10.87% across species, configurations, and future projections. Differences in thresholded suitable area, calculated as unclamped minus clamped, ranged from −0.74 to +26.04 km^2^. Thus, small domain-average differences did not preclude localized effects or changes in thresholded area. These results apply to the deterministic full-data diagnostic models and do not quantify clamping effects on the complete bootstrap ensemble (Table S8).

### 2.8. GCM and Bootstrap Variation in Projected Outcomes

The ensemble-map changes were not uniformly shared by the individual GCMs (Table S10). Under the nine-variable 10P configuration and SSP5-8.5 in the 2070s, *B. faberi* suitable area was 0.00, 0.74, and 1885.53 km^2^ for BCC-CSM2-MR, MIROC6, and MPI-ESM1-2-HR, respectively, compared with 435.83 km^2^ currently. The corresponding areas for *Y. brevipaniculata* were 0.00, 986.05, and 2292.42 km^2^, compared with 1052.92 km^2^ currently. Thus, each ensemble-map expansion was accompanied by one GCM-specific increase and two decreases. Under the nine-variable and DEM-containing five-variable production configurations, *C. szechuanensis* contracted in the ensemble-mean maps, although its nine-variable MPI-ESM1-2-HR projection under SSP1-2.6 in the 2070s increased by 5.99% at 10P, and additional small increases occurred under the five-variable configuration. In the BIO1-containing temp5 diagnostic, its ensemble-mean suitable area increased at 10P in all four future scenarios (Section 2.5; Table S13). A zero suitable-area result denotes absence above the specified threshold, not observed extinction.

Aggregation order also affected the numerical outcomes and sometimes their change direction. For *B. faberi* under the five-variable 10P configuration and SSP5-8.5 in the 2070s, the arithmetic mean of the three GCM-specific suitable areas was 729.60 km^2^, above the current 598.05 km^2^, whereas thresholding the ensemble-mean surface gave 492.51 km^2^, a decrease. For the combined resource under the nine-variable 10P configuration, PLAND, AREA_MN, and AI each increased under one GCM and decreased under two in all four future scenarios. Under SSP5-8.5 in the 2070s, mean GCM-specific AREA_MN was 4180.25 ha, above the current 2297.53 ha, whereas the ensemble map gave 2112.50 ha. Pairwise overlap also varied among GCMs: *B. faberi*–*Y. brevipaniculata* D decreased under all three GCMs in the first three scenario–period combinations but increased under one and decreased under two in the late-century SSP5-8.5 projection, under both predictor configurations.

Within-species bootstrap comparisons retained important GCM differences. For *B. faberi* at 10P under SSP5-8.5 in the 2070s, all ten paired repetitions decreased under BCC-CSM2-MR and MIROC6 and increased under MPI-ESM1-2-HR, in both configurations. For nine-variable *Y. brevipaniculata* under the same scenario, MIROC6 produced four increases and six decreases, whereas BCC-CSM2-MR produced ten decreases and MPI-ESM1-2-HR ten increases. Repetition-level landscape summaries retained undefined values where no suitable cells or too few patches remained. These are empirical sensitivities conditional on the retained samples and fixed thresholds, rather than confidence intervals or independent validation.

Spatial agreement maps identify where individual GCMs project gains or losses relative to the common current ensemble map (Figures S1 and S2). In the nine-variable 10P projection for SSP5-8.5 in the 2070s, no newly suitable *B. faberi* or *Y. brevipaniculata* cells were supported by all three GCMs. Unanimous losses covered 29.65 km^2^ for *Y. brevipaniculata* and 76.69 km^2^ for *C. szechuanensis*. Agreement counts describe the selected GCMs, not probabilities of future occupancy.

### 2.9. Elevation Bands and Candidate Monitoring Areas

Elevation-band summaries located the projected changes more precisely within the modeling domain (Table S16). At 10P, the nine-variable ensemble projected net losses of *Chimonobambusa szechuanensis* of 23.83–100.43 km^2^ at 1500–<2000 m and 18.67–59.69 km^2^ at 2000–<2500 m across the four future scenario–period combinations. The corresponding losses under the DEM-containing five-variable configuration were 106.37–142.83 and 50.74–62.68 km^2^. In these two configurations, 90.35–97.08% of the area where all three GCMs agreed on loss in each scenario–period combination lay between 1500 and <2500 m. These are changes in thresholded predictions, not measured bamboo losses.

For *C. szechuanensis* at 10P, cells with unanimous GCM loss in both original configurations covered 10.40, 8.91, 40.98, and 39.48 km^2^ under SSP1-2.6 in the 2050s and 2070s and SSP5-8.5 in the 2050s and 2070s, respectively. Twelve cells, totaling 8.91 km^2^, met this condition in all four combinations; eight of them (5.94 km^2^) were at 1500–<2000 m. Requiring agreement with the BIO1-containing configuration reduced the scenario-specific intersections to 2.97, 2.97, 3.71, and 0.00 km^2^, with no cells shared across all four combinations. Under the BIO1-containing ensemble, net suitable area instead increased by 7.38–106.97 km^2^ at 1500–<2000 m and 93.07–239.79 km^2^ at 2000–<2500 m. Figure S4 maps GCM loss agreement separately for all three configurations. These contrasts identify conditional monitoring candidates and areas of model disagreement, rather than configuration-independent risk zones.

## 3. Discussion

### 3.1. Environmental Associations and Species-Specific Responses to Climate Change

The three species differed in predictor contributions and marginal responses under common model settings. Precipitation of the wettest quarter contributed most to the *Bashania faberi* model; precipitation of the wettest month and elevation were prominent for *Yushania brevipaniculata*. For *Chimonobambusa szechuanensis*, wettest-quarter and driest-month precipitation contributed substantially, while predicted suitability decreased as mean diurnal temperature range increased. These differences are consistent with reported variation in bamboo populations and distributions across environmental gradients [7,8], associations between panda habitat use and vegetation or topography [9], and species-specific climate projections [10,11,12,13]. However, predictor correlations, background extent, sample distribution, and model settings jointly influence MaxEnt contributions. These values therefore describe predictive associations within the fitted models, not independent ecological effects.

The direct elevation diagnostic illustrates this dependence on conditioning (Figure S5): at 1000–<1500 m, the precipitation means used in the curves lie below the corresponding grid-cell ranges, whereas predictions under actual environmental combinations peak at intermediate or higher elevations. The negative elevation-related terms explain the conditional decline within these fitted models. They do not demonstrate that elevation independently limits either species or that low-elevation sites are preferred; the occurrence data and their sampling limitations remain unchanged.

Responses also varied with the study system and scale. At the baseline 10P thresholds, suitable areas for *B. faberi* and *Y. brevipaniculata* were projected to expand only under SSP5-8.5 in the 2070s, whereas *C. szechuanensis* declined in every scenario. Previous studies projected an expansion in aggregate bamboo community area in Sichuan [12], the contraction of a primary food bamboo in the Qinling Mountains [13], and contraction for *Bashania fangiana* but expansion for *Yushania brevipaniculata* across a broader region [11], using the original name for the taxon treated as *A. faberi* in Flora of China [16]. These studies differ in spatial scale, species assemblage, occurrence records, background extent, predictors, climate inputs, and thresholds, which limits direct comparison of their projections.

Species-level expansion need not increase the combined suitable area. Under SSP5-8.5 in the 2070s, baseline 10P areas increased for *B. faberi* and *Y. brevipaniculata*, yet combined PLAND declined from 34.20% to 31.05%. Their areas at the higher fixed thresholds also contracted. Resource interpretation must therefore distinguish individual from combined responses and lower-threshold expansion from changes in areas with higher predicted suitability.

Warming experiments provide complementary evidence on survival and resource quality. Yang et al. [14], studying *Bashania fangiana*, identified a possible survival threshold between 1.5 and 3 °C warming; stronger warming also reduced nutritional value and increased aphid damage. Zhang et al. [15], studying *Bashania faberi*, found increased subsurface soil nitrate nitrogen and leaf nitrogen content under 1.5 °C warming, but reduced survival and aboveground biomass under 3–4.5 °C warming as the experiment continued. These studies use alternative names placed in synonymy under *A. faberi* by Flora of China [16]. Such results show that climatic suitability captures only part of resource risk: biomass, regeneration, nutritional quality, pests, and understory conditions also influence actual bamboo resources.

Differences in nutritional composition among bamboo species and plant parts [2,3,4,6], together with differences in aboveground biomass [5], mean that the expansion of one species cannot be assumed to compensate for the loss of another. Assessing such compensation would require measurements of biomass, nutrition, regeneration, and panda use alongside the suitability projections.

### 3.2. Uncertainty Associated with Climate Models, Thresholds, and Variable Combinations

Averaging across GCMs reduces dependence on a single climate model, and the standard deviation among them describes disagreement in their projections [23,24]. This disagreement was greatest for *Yushania brevipaniculata*, intermediate for *Bashania faberi*, and lowest for *Chimonobambusa szechuanensis*. For the first two species, disagreement peaked under SSP5-8.5 in the 2070s, the scenario in which the baseline 10P analysis projected expansion. Even where the three GCMs agree, their agreement does not independently validate predictive accuracy or account for uncertainty in sampling, parameters, or predictors. Calculating outcomes separately for each GCM revealed direction disagreements obscured by the ensemble maps. In particular, the two late-century baseline expansions were not supported by a majority of the selected GCMs. Averaging suitability, thresholding, identifying patches, and normalizing overlap are different operations whose order can change the result; the ensemble-map estimate and the mean of GCM-specific outcomes should therefore be reported as distinct summaries (Table S10).

Threshold choice affected even the direction of projected area change. The baseline ensemble expansion of *B. faberi* and *Y. brevipaniculata* under SSP5-8.5 in the 2070s became contraction at fixed thresholds of 0.5 and 0.6, whereas the *C. szechuanensis* ensemble maps declined at all three thresholds. This agrees with evidence that binarization influences predicted ranges and ecological interpretation [25,26,27] and underscores the need to identify threshold-dependent conclusions.

Changing to the five-variable configuration also reversed the two baseline 10P expansion results. Using the original future climate products, ensemble-mean contraction of *C. szechuanensis* and declines in combined PLAND, AREA_MN, and AI persisted under the nine-variable and DEM-containing five-variable configurations. These patterns do not extend to every predictor configuration or model setting. The separate GCM analysis showed that these ensemble responses were not universal across climate models, including exceptions to *C. szechuanensis* contraction.

The additional feature-class and regularization comparison partly addresses model-complexity uncertainty. Mean AUCs were broadly similar, but omission rates and the number of active lambda terms varied; no setting produced a uniform direction of future area change across all GCM, scenario, and period combinations. We retain LQP RM = 2 as the original production baseline and report the alternative configurations separately. This comparison does not establish that this setting is optimal, particularly for *B. faberi* with only 15 occurrence records.

The environmental-novelty overlay shows that the late-century gains for *B. faberi* and *Y. brevipaniculata* include substantial areas outside the calibration environmental ranges in projections from at least one GCM. These gains should therefore be interpreted as conditional projections rather than evidence of secure expansion. Likewise, low inter-GCM standard deviations for *C. szechuanensis* do not exclude environmental extrapolation. Clamping produced localized changes in the full-data diagnostic fits despite small domain-average differences, reinforcing the need to assess environmental support alongside agreement among climate models.

The fixed thresholds of 0.5 and 0.6 identify levels of cloglog prediction, not independently validated habitat quality classes. With only 15 valid *B. faberi* records, local boundaries and patch patterns require further assessment of sampling coverage and validation performance using additional survey data; a single scenario or threshold cannot support precise site-level inference.

### 3.3. Changes in Landscape Structure, Spatial Relationships Among Bamboo Species, and Structural Continuity

Combined PLAND, AREA_MN, and AI declined in the baseline ensemble analysis even though NP changed in different directions across scenarios. Suitable area and mean patch size can therefore decrease without an increase in patch number. AREA_MN equals total suitable patch area divided by NP, so a decline in AREA_MN is partly dependent on changing suitable area and is not independent evidence of increased fragmentation. Similar differences between area and fragmentation trends have been reported elsewhere [21,22], supporting the use of patch attributes alongside suitability predictions [28]. The approximately 1 m increase in ENN_MN under SSP1-2.6 in the 2070s was too small, by itself, to provide clear evidence of increased isolation.

With the original future climate products and the two original predictor configurations, combined PLAND and AI declined across all three thresholds on the 1 km grid and AREA_MN declined under both patch definitions. At the 5 km grain, the three metrics declined at 10P, but higher thresholds produced increases in some AREA_MN or AI values and undefined metrics where no suitable cells remained. The baseline increase in combined LPI under SSP5-8.5 in the 2070s became a decrease on the coarse grid, and NP responses also changed with grain. Declining combined suitable extent persisted within these settings, but patch size, aggregation, and isolation depended on threshold and grain. Within each grain, all species and scenarios shared a mask derived from the original domain; representing that mask on different grids changed its area. Aggregating predictions tests output-grid sensitivity and does not fully separate input-climate resolution effects from climate-change effects. Moreover, even at the baseline 10P threshold and 1 km grain, the GCM-specific combined-resource PLAND, AREA_MN, and AI changes were not unanimous. Robustness to a particular processing choice should not be equated with robustness to climate-model choice.

The additional input-product comparison confirms that matching nominal climate-grid resolution does not make landscape conclusions invariant (Table S17). Species-level ensemble area directions at 10P persisted, but the nine-variable late-century *Y. brevipaniculata* expansion became smaller, and the corresponding combined AI change reversed from a decline to a very small increase. Higher thresholds produced additional reversals under the BIO1-containing configuration. These results require explicit qualification by the climate product in addition to GCM, predictor configuration, threshold, and output grain. A finer downscaled product is not an independent fine-scale observation and should not be assumed more accurate solely because its grid is finer.

Individual species contributed differently to these combined patterns. Under SSP5-8.5 in the 2070s, baseline PLAND, AREA_MN, and AI increased for *Bashania faberi*. For *Yushania brevipaniculata* under the same scenario, PLAND increased, whereas AREA_MN and AI declined. For *Chimonobambusa szechuanensis*, PLAND and AREA_MN declined in every scenario, although AI increased slightly under SSP1-2.6. The combined pattern thus cannot be interpreted as uniform deterioration in every species and metric.

Richness and Schoener’s *D* describe additional spatial relationships. Suitable bamboo species richness ranged from 0 to 2, with no pixels meeting all three species-specific 10P thresholds. Under the baseline configuration, D increased for both pairs involving *C. szechuanensis*, remaining low for its overlap with *B. faberi*, while the *B. faberi*–*Y. brevipaniculata* response varied by scenario. These differences resemble previously reported asynchronous pairwise overlap changes [20] but do not demonstrate field coexistence or dietary substitution.

Higher D also need not indicate an improvement in overall resource conditions. Under SSP5-8.5 in the 2050s, D for *Y. brevipaniculata* and *C. szechuanensis* increased from 0.3288 to 0.4806 while combined PLAND, AREA_MN, and AI declined. Greater similarity between normalized suitability surfaces can therefore coexist with reduced suitable extent, mean patch size, and aggregation.

Structural continuity here describes the area, aggregation, and isolation of predicted suitable patches; it does not measure functional connectivity for giant panda movement. The projected declines in some aspects of this structure raise concerns for potential resources. Assessing their ecological consequences requires information on actual bamboo stands, panda activity, and local environmental conditions.

### 3.4. Implications for Conservation and Management

Projected contraction areas warrant monitoring, particularly for *Chimonobambusa szechuanensis*, whose declines persisted under the original nine-variable and DEM-containing five-variable production configurations across all three thresholds and four scenarios. By contrast, the BIO1-containing temp5 diagnostic projected an increase in its ensemble-mean suitable area at 10P in all four scenarios (Table S13). For *Bashania faberi* and *Yushania brevipaniculata*, the ensemble-map expansion under SSP5-8.5 in the 2070s depended on thresholds and predictors. Individual GCMs also differed in their projected change directions for all three species. Surveys of distribution, regeneration, and habitat conditions are needed before interpreting those projections as resource recovery. Scenario planning and long-term monitoring can then be updated as regional field information becomes available [29,30].

The 1500–<2500 m band provides a candidate stratum for monitoring *C. szechuanensis* within the modeled domain: it contains most of the unanimous GCM losses under the two original configurations (Table S16; Figure S4). Sites where those configurations agree on loss can be paired with sites where the BIO1-containing configuration gives a contrasting outcome, allowing field observations of persistence, regeneration, and resource quality to inform subsequent model evaluation. The absence of an all-configuration, all-scenario loss intersection precludes describing these candidates as universally robust priority zones. Their designation is model-derived, does not certify occurrence or future loss at a site, and does not establish statutory national-park management boundaries. These candidates are based on the original future climate products; their spatial agreement was not recomputed under the alternative 30-arc-second product (Table S17).

**Figure 8:**
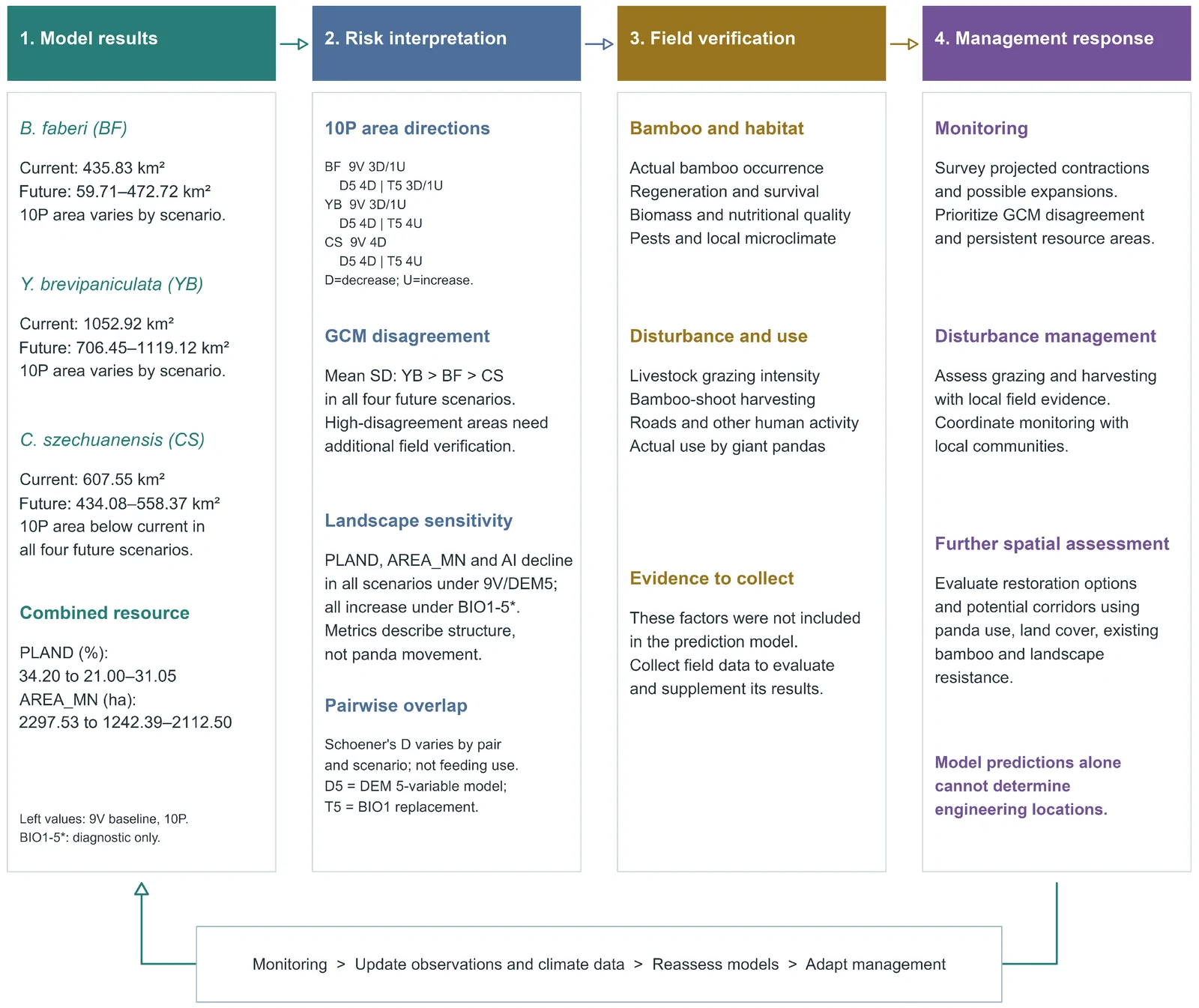
Framework for assessing risks to potential food resources across bamboo species and informing adaptive management under climate change in the modeled Qionglai Mountains study area. Model results, risk interpretation, field verification, and management responses form a feedback loop through monitoring, data updates, model reassessment, and management adjustments. Numerical values represent the nine-variable baseline with species-specific 10P thresholds and the original 2.5-arc-minute future products; future ranges span the four ensemble scenarios. Combined suitable-area values are shown as the current value followed by the future range. BF, YB, and CS denote *Bashania faberi*, *Yushania brevipaniculata*, and *Chimonobambusa szechuanensis*, respectively. Predictor configurations are 9V (nine-variable baseline), D5 (DEM-containing five-variable configuration), and T5 (BIO1-containing temp5 diagnostic). The baseline 10P expansions for *B. faberi* and *Y. brevipaniculata* under SSP5-8.5 in the 2070s depend on predictor configuration; *C. szechuanensis* declines under 9V and D5 but increases under T5 across all four ensemble scenarios. The asterisk (*) identifies BIO1-5 as a diagnostic sensitivity configuration, not a statistical significance test. Arrows between the four stages indicate the proposed assessment sequence; the return arrow represents feedback from monitoring and updated observations to model reassessment. T5 is a sensitivity configuration; see Section 2.5 and Table S13 for numerical comparisons and Table S17 for climate-product sensitivity and its exceptions. Field verification and management responses are proposed activities: this study did not conduct corresponding field validation, functional-connectivity assessment, or intervention-site selection. GCM, global climate model; SD, standard deviation; PLAND, percentage of landscape; AREA_MN, mean patch area; AI, aggregation index; NP, number of patches; SSP, Shared Socioeconomic Pathway; 10P, 10th-percentile presence threshold.

The combined-area results suggest that surveys should assess large patches, aggregation, and spatial relationships among bamboo species alongside area and patch number. Agreement across tested thresholds can help set survey priorities; disagreement among GCMs or predictor configurations identifies results that need further assessment. Figure 8 links these findings to possible management responses. The field verification, resource-quality assessment, and corridor analysis shown there are proposed follow-up activities, none of which was completed as validation in this study. Applying these regional monitoring candidates within the Giant Panda National Park would require a documented boundary overlay and field verification.

Livestock grazing and bamboo shoot harvesting may additionally affect resources, but neither was modeled here, so projected suitability changes cannot be attributed to these disturbances. In Wolong, grazing affected clonal populations of *B. faberi* more strongly than wild panda foraging, suppressing young bamboo emergence, regeneration, and growth and reducing bamboo-layer cover [31]. Where grazing is confirmed within projected risk areas, monitoring should jointly assess grazing intensity, regeneration, and population structure to inform local management. Harvesting pressure also varies among mountain ranges, with prolonged harvesting periods reported in parts of the Qionglai Mountains [32]. Where field evidence indicates substantial pressure, locally appropriate responses may include limits on harvest quantities and periods, outreach, and reduced dependence on wild shoots [32].

For restoration and corridor planning, previous work combined least-cost paths derived from panda habitat suitability and landscape resistance with food bamboo projections to identify candidate species and planting areas [33]. Our projections can support preliminary screening for such work, but choosing intervention sites requires information on actual panda use, existing bamboo, land cover, disturbance, and resistance (Figure 8).

### 3.5. Study Limitations and Future Directions

We retained the original production setting of RM = 2 with LQP features and added a limited diagnostic comparison of feature classes and RM values. The alternatives produced broadly similar cross-validated discrimination but did not yield a uniform direction of future area change across the tested GCM, scenario, and period combinations; because we tested a limited set of configurations without formal model selection, model-complexity uncertainty remains.

Combined PLAND, AREA_MN, and AI declined at 10P when ensemble-mean predictions were aggregated to 2.5 arc-minutes and landscape metrics were then calculated on a 5 km equal-area grid, but fixed higher thresholds produced exceptions for AREA_MN and AI or left them undefined. Patch delineation also affected absolute patch counts and mean patch areas. The 1 km metrics, especially those for *B. faberi*, should therefore be interpreted as comparative, indicative summaries rather than precise patch boundaries or resolved functional connectivity. This output-grain test is complemented by the separate future climate product comparison.

The 30-arc-second future-product diagnostic matches the nominal baseline climate resolution while retaining the fitted models (Table S17). Matching grid resolution does not establish equivalent accuracy or resolve sub-grid climate. Its landscape comparison used the 1 km grid and eight-neighbor rule; the full grain-by-connectivity analysis, elevation-band summaries, and monitoring-agreement map were not recomputed for this new product. Consequently, Table S16 and Figure S4 remain conditional on the original 2.5-arc-minute future climate products and cannot be treated as product-independent monitoring priorities.

The fold-level validation results illustrate why the small *B. faberi* sample requires cautious interpretation. Under the original nine-variable full-domain-background design, omission was 100%, 50%, 0%, and 0% across its four folds (4/4, 2/4, 0/4, and 0/3), yielding a pooled omission rate of 40.00% and an arithmetic mean rate of 37.50%. Refitting with geographically partitioned backgrounds reduced pooled omission to 33.33%, but fold 1 still omitted all four test records despite an AUC of 0.9407. A high AUC therefore does not establish acceptable threshold-based prediction or stable local boundaries. Environmental-range differences describe potential limitations in coverage but do not by themselves explain the failures. All designs reuse the same small occurrence dataset; none adds independent external records. We consequently treat *B. faberi* distribution boundaries and landscape metrics as indicative and avoid site-specific inference.

High correlations remained among baseline predictors, so their percent contributions cannot be interpreted as independent ecological effects. The low-correlation configuration improved some spatial validation metrics, but omission rates remained high in some folds and suitable-area changes reversed direction for particular species and scenarios. The reduced-predictor comparison used the stated correlation-based rule; the separate regularization and background diagnostics were also limited rather than an exhaustive model search. Relative to the original full-domain-background validation, geographically partitioned background fits and evaluations produced species-dependent changes in discrimination and omission, with some fold failures persisting (Table S11). None of these comparisons identifies an optimal model. The complete historical sequence of decisions selecting the original nine predictors from 22 candidates, including the reason for excluding absolute-temperature predictors, has not been recovered. The BIO1-containing sensitivity analysis examines dependence on predictor choice but does not reconstruct those historical decisions. Independent occurrence records are still needed to assess spatial prediction and transferability across climatic conditions.

Terrain preprocessing introduces further uncertainty. The production models retained angular aspect, and direct angular interpolation differed substantially from circular-component interpolation at some model-grid centers. Separate aspect and slope sensitivity fits changed validation summaries but did not resolve the omission limitations for *B. faberi* (Table S15). The slope reconstruction supports numerical consistency with a horizontal unit conversion, without recovering the complete historical processing environment. The historical DEM resampling settings and treatment of flat cells remain incompletely documented. The aspect comparisons also increased the predictor count by one, so representation and model-feature changes cannot be separated. Neither diagnostic was propagated to future suitability maps, suitable-area estimates, or landscape metrics. Their validation results therefore do not establish the stability of those downstream outcomes.

The 2025 occurrence records also differ in temporal coverage from the 1970–2000 climatic baseline. Thus, “current” describes predictions under historical climate, not observed conditions or bamboo resources in 2025. Long-term mean climate variables also omit extremes such as severe drought and high temperatures. For 189 *Rhododendron* species in China, adding extreme-climate indicators substantially altered future suitable-area projections, with drought and high-temperature extremes strongly influencing predictions for most species [19]. Their effects on the three bamboo species remain unknown and warrant testing. Regional models can also overestimate or underestimate climate risks for local panda populations [30], so applying these projections to specific sites requires field verification.

MESS assesses environmental similarity relative to the modeled background domain, not the environmental coverage of the occurrence sample alone. Non-negative MESS does not establish that joint predictor combinations are well represented or that transferred predictions are accurate. The clamping comparison used full-data models and did not propagate clamping effects through all bootstrap replicates. These diagnostics therefore qualify the transferability of the projections without providing independent validation, or evidence of actual occupancy, dispersal, regeneration, or bamboo availability.

The outcome-level GCM and bootstrap analyses characterize selected sources of uncertainty rather than total predictive uncertainty. Three selected GCMs do not define a probabilistic distribution of future climate, and ten resamples of the existing occurrences cannot correct survey bias or add environmental coverage. The bootstrap comparisons hold mapping thresholds fixed and therefore exclude threshold-estimation uncertainty. Cross-species resample numbers have no established paired sampling design; pairwise D sensitivity uses all 100 combinations under an explicit independence assumption, while a joint bootstrap distribution for combined-resource landscape metrics was not estimated. Bootstrap landscape comparisons were restricted to the primary 10P threshold and 1 km grain. These limits also preclude interpreting agreement maps or empirical ranges as probabilities or confidence intervals.

Survey sites were selected purposively using habitat and environmental information, and routes mainly followed existing forest roads and patrol trails. This design may produce uneven survey effort across the modeling domain; the retained occurrence archive does not quantify search effort or the inclusion probability of surveyed sites. GPS tracks and plot-level habitat records are available, but quantitative survey-effort coverage and inclusion probabilities have not been incorporated into the reported analyses. Removing repeated records within cells therefore does not establish representative coverage of the modeling domain. The spatial-background diagnostics in Table S11 partition this same domain; they do not test a survey-effort correction or independently justified species-specific accessible areas. These sources of sampling and background-domain uncertainty remain unquantified.

Further surveys over multiple years, particularly for *B. faberi*, could broaden environmental coverage and provide independent records for validation. Broader comparisons of RM values and feature-class combinations, beyond the limited sensitivity analysis reported here, would help further assess model-complexity uncertainty [34]. Where data permit, subsequent models should incorporate extreme-climate indicators and field information on soil, microenvironment, bamboo regeneration and resource quality, disturbance, and actual use by pandas. Field assessments of habitat quality, Global Positioning System (GPS) tracking, and bamboo monitoring have been proposed to refine local climate-risk assessments [30]. These data would help evaluate what the projected suitability changes mean for bamboo resources and panda habitat.

## 4. Materials and Methods

### 4.1. Study Area

**Figure 9:**
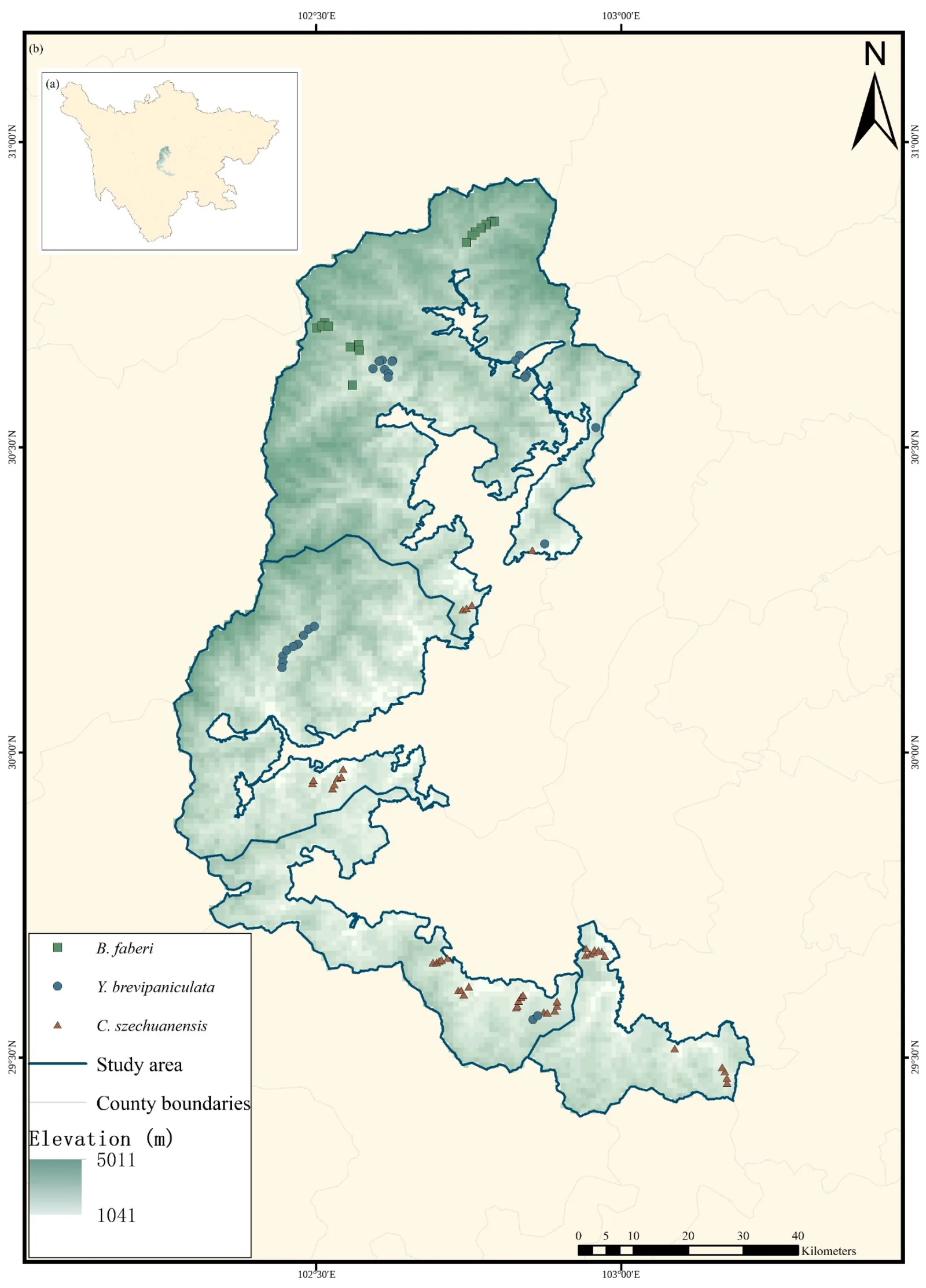
Location of the study area in Sichuan Province and occurrence records of the three food bamboo species. (**a**) Location of the study area within Sichuan Province; (**b**) topography of the study area and occurrence points of *Bashania faberi*, *Yushania brevipaniculata*, and *Chimonobambusa szechuanensis*. The points shown are the valid occurrence records used for MaxEnt modeling after field survey records were deduplicated within a 30-arc-second geographic grid, checked for environmental data validity, and further deduplicated within environmental raster cells. Base-map colors indicate elevation (m). The displayed topographic background raster spans 1041–5011 m; the common valid environmental modeling domain used for MaxEnt spans 1063–4902 m in the operative environmental DEM. These ranges refer to different raster value domains. The inset is panel (**a**) and the main topographic map is panel (**b**). The location-map background in panel (**a**) was derived from *The Pandas of Sichuan: The 4th Survey Report on Giant Panda in Sichuan Province* and georeferenced in ArcGIS 10.8.2. It provides cartographic context and does not establish a verified statutory park boundary.

We modeled the potential suitability of three food bamboo species within a defined study area in the Qionglai Mountains region of Sichuan, China, with the analysis encompassing parts of Baoxing, Tianquan, Yingjing, and Hongya counties. The study area and occurrence records of the three bamboo species are shown in Figure 9, and the extent of the environmental domain used for modeling is described below. Previous regional research has identified Baoxing and Tianquan as important giant panda distribution areas in the central Qionglai Mountains, with Yingjing and Hongya located in adjacent areas to the south [10]. The Qionglai Mountains and adjacent ranges have pronounced topographic relief and distinct elevational zonation of forest vegetation associated with mountain climatic conditions [9,10].

The environmental domain used for modeling comprised the area with valid data for all nine environmental variables used in MaxEnt. It contained 7333 valid grid cells at a spatial resolution of 30 arc-seconds, with geographic bounds of 102.2333–103.2250° E and 29.3833–30.9500° N and elevations ranging from 1063 to 4902 m. These grid-cell counts, geographic bounds, and elevation ranges refer exclusively to the environmental analysis domain used in this study. They neither correspond to the complete administrative extents of Baoxing, Tianquan, Yingjing, and Hongya counties nor represent the official area or full statutory boundaries of the relevant portions of the Giant Panda National Park. The same operational domain was used for all three species to provide a common regional comparison. Its valid-data mask was not independently delineated as the area accessible to each species. All reported areas and landscape metrics refer to this domain; they are not park-wide totals or measurements restricted to a verified statutory park extent.

The archived study-area polygon was spatially consistent with the joint valid-data mask at the grid-cell-center level: all 7333 valid environmental cell centers fell within the polygon, and no additional grid cell centered within the polygon lacked complete predictor values on the retained template. This comparison documents the relationship between the archived study-area polygon and the operational modeling domain. It does not authenticate the polygon as the statutory national-park boundary or reconstruct the complete historical masking workflow. The location-map background in Figure 9a was derived from *The Pandas of Sichuan: The 4th Survey Report on Giant Panda in Sichuan Province*, compiled by the Sichuan Provincial Forestry Department, and georeferenced in ArcGIS 10.8.2. The georeferenced map provides cartographic context and is not treated as a verified statutory park boundary for park-specific area calculations.

Previous studies have documented abundant bamboo resources in the Qionglai Mountains and neighboring giant panda distribution areas, where *Bashania faberi*, *Yushania brevipaniculata*, and *Chimonobambusa szechuanensis* are important food bamboo species for giant pandas [5,10]. Field surveys conducted for this study also recorded distinct spatial distributions of the three species within the study area. The pronounced elevational gradient and spatial differentiation among these bamboo species provide a setting for comparing their potential suitability and responses to future climate under a common set of environmental variables and a unified modeling framework.

### 4.2. Species Occurrence Data and Environmental Variables

The final modeling records of *Bashania faberi*, *Yushania brevipaniculata*, and *Chimonobambusa szechuanensis* span distinct DEM-derived elevation ranges (Table 7). Field surveys were conducted from October to December 2025 to verify occurrence locations, guided by distribution information from materials associated with the Fourth National Giant Panda Survey in Sichuan Province, published studies, and records from the Chinese Virtual Herbarium. All occurrence records ultimately used for modeling were verified in the field.

Survey sites in Hongya, Tianquan, Baoxing, and Yingjing counties were selected purposively using spatial overlays in ArcGIS 10.8.2. Habitat-distribution information from the Fourth National Giant Panda Survey and food-bamboo distribution information from an illustrated atlas were considered together with soil, geological, and vegetation maps; DEM-derived elevation; slope and aspect; and remotely sensed NDVI. Areas were selected by considering the concentration of potential bamboo habitat and reported giant panda distribution density. Routes mainly followed existing forest roads and patrol trails, extending from valleys toward ridges to traverse different vegetation belts and slope aspects. Field sampling combined standard plots with reconnaissance occurrence records. In core areas with extensive, continuous stands of target bamboos, 20 m × 20 m forest plots were established, each containing three 1 m × 1 m bamboo subplots in a triangular arrangement. Within each bamboo subplot, bamboo culms were counted manually, basal diameter was measured using a vernier caliper, bamboo height was measured using a measuring tape, and bamboo cover was estimated visually. For scattered patches and patches at distribution margins encountered during reconnaissance, temporary plots were used to verify occurrences. A handheld GPS was used to record coordinates and elevation at standard plots and reconnaissance sites; slope, aspect, and tree-canopy closure were also recorded at survey plots. Occurrence records from both sampling components were pooled before data cleaning and deduplication. December fieldwork included quadrat sampling, specimen collection, and field-data recording.

GPS tracks of the field survey routes were retained, and canopy closure and bamboo cover were recorded at the survey plots. All collected bamboo specimens were identified by Prof. Peihao Peng through the comparison of morphological characters with taxonomic references, including *Flora Reipublicae Popularis Sinicae*. Collection numbers, dates, and localities were recorded for the specimens, which are retained in the plant laboratory, College of Geography and Planning, Chengdu University of Technology. The original plot forms recording canopy closure and bamboo cover are retained there as paper records and have not been digitized. The available archive contains 34 GPS tracks recorded during the survey campaign, with date labels from 15 October to 17 November 2025. The tracks were not classified into active field-search and transit segments; consequently, total track length was not used to quantify survey effort. The 268 initial records of the three study species exactly match point geometries in the supplied bamboo survey layer, and all 83 final modeling records match this layer within 1 × 10^−6^ degrees.

The initial dataset comprised 30 occurrence records for *B. faberi*, 107 for *Y. brevipaniculata*, and 131 for *C. szechuanensis*. To reduce the influence of spatial clustering on model results, a fishnet grid with a spacing of 0.008333° (approximately 30 arc-seconds) in both longitude and latitude, together with its cell center points, was created in ArcGIS 10.8.2. Spatial Join was used to associate occurrence points with the nearest grid center (JOIN_ONE_TO_ONE, CLOSEST; search radius: 0.01°), and the center-point identifier field FID1 was mapped to Grid_ID. Delete Identical was then applied using the combination of species and Grid_ID to retain one point per species–grid combination, yielding 17 records for *B. faberi*, 35 for *Y. brevipaniculata*, and 51 for *C. szechuanensis*. A record-level audit reconstructed all 103 species–grid groups from the archived point geometries and fishnet labels. In every group, the retained TARGET_FID was the smallest FID in the archived source table, and its geometry was identical to that source feature.

The retained records were then checked against the environmental rasters used for modeling. Two *C. szechuanensis* records lacking valid environmental values were excluded, and only one record was retained where multiple records of the same species fell within the same environmental raster cell. The final dataset contained 15, 25, and 43 valid occurrence records for *B. faberi*, *Y. brevipaniculata*, and *C. szechuanensis*, respectively. This procedure removed duplicate records within geographic grid cells and environmental raster cells; it did not guarantee a minimum distance of 1 km between retained points. For the subsequent environmental-cell deduplication, all 83 final records matched the first valid record in their species–cell group in the archived fishnet-output order; coordinate differences were at most 5.1 × 10^−8^ degrees.

DEM-derived elevations at the final occurrence locations are summarized in Table 7, and their marginal environmental predictor ranges are provided in Supplementary Table S14. Elevations are extracted from the environmental raster and are not field measurements. The record-level audit identifies the retained records at both deduplication stages. It does not establish why the source records had that order or demonstrate a preference based on observation date or coordinate accuracy. Survey routes, search effort, and representative survey coverage cannot be inferred from the retained records. To describe the effect of record processing on environmental coverage, we extracted the nine predictor values at the initial survey records and compared occupied environmental cells and marginal ranges with those of the final records (Table S14). Range comparisons used only records complete for all nine predictors and excluded aspect because of its circular scale. Initial medians include repeated records within environmental cells; these summaries describe occurrence support rather than search effort or sampling representativeness.

The name *Bashania faberi* (Rendle) T. P. Yi is retained throughout our analyses. Flora of China [16] lists this name and *B. fangiana* (A. Camus) P. C. Keng & T. H. Wen as synonyms of *Arundinaria faberi* Rendle. We retain the names used by Li et al. [11], Yang et al. [14], and Zhang et al. [15] when discussing their studies.

The initial set of candidate environmental predictors comprised 19 bioclimatic variables and three topographic variables. Bioclimatic variables were obtained from WorldClim 2.1 for the baseline period of 1970–2000 at a spatial resolution of 30 arc-seconds [35]. Throughout this study, “current” refers to predictions under this historical climatic baseline. The topographic variables were elevation, slope, and aspect. Elevation data were obtained from the Advanced Spaceborne Thermal Emission and Reflection Radiometer Global Digital Elevation Model (ASTER GDEM) at 30 m resolution. Slope and aspect were derived from the processed elevation data, and all three topographic predictors were aligned to the 30-arc-second bioclimatic grid. The recovered processing history and new numerical checks are described below. A common nine-variable baseline configuration was used, comprising elevation (dem), slope (slope), aspect (aspect), mean diurnal temperature range (bio02), annual precipitation (bio12), precipitation of the wettest month (bio13), precipitation of the driest month (bio14), precipitation of the wettest quarter (bio16), and precipitation of the driest quarter (bio17). Because this set included some highly correlated predictors, an additional low-correlation configuration was established to assess the influence of variable combinations on model predictions.

For ecological interpretation, the precipitation predictors describe annual precipitation and wet- and dry-period conditions [35]. We treat these as descriptors of the moisture environment, not as direct measurements of soil water or independently established physiological limits. BIO2 describes the mean diurnal temperature range rather than absolute temperature. Elevation, slope, and aspect describe terrain position, inclination, and orientation; they are spatial covariates, not substitutes for future temperature change. The BIO1-containing comparison therefore tests the influence of adding an explicit annual-mean temperature dimension. These interpretations explain the environmental dimensions represented by the predictor configurations; they do not reconstruct the undocumented historical choice of the original nine variables.

Archived geoprocessing records indicate that slope and aspect were derived via a fine-resolution geographic grid before bilinear resampling to the 30-arc-second grid (Table S15). The production models used aspect in angular degrees. The 7333 valid modeling cells contained aspect values of 0.136308–358.382904° and no −1 flat-terrain codes. However, 107 cells in the archived fine-resolution aspect raster were coded as −1; a separate record documenting their treatment before historical resampling was not recovered. The records establish the processing order but do not fully establish the effective terrain-derivation environment or the DEM resampling procedure.

The archive confirms a preliminary run containing 22 candidate predictors and exploratory contribution-assisted correlation-screening code. However, the available files do not establish a complete historical sequence of decisions that produced the nine predictors used in the production models. We therefore do not present a reconstructed 22-to-nine procedure as historical fact; recovered records and new diagnostics are distinguished in Supplementary Table S12. The retained nine-variable baseline contains 12 predictor pairs with absolute Pearson correlations above 0.8 across the jointly valid background cells; it is not presented as a set satisfying that correlation threshold.

The archived DIANWEI table was audited separately from the modeling inputs (Table S12). Its geoprocessing metadata records the extraction of 22 environmental rasters to points on 23 March 2026. Two of its 103 intermediate records contained −9999 in all 22 fields; these values were treated as missing for the correlation audit. All 83 final modeling occurrences matched valid intermediate records, and neither missing-value record was retained. Archived training inputs and logs for 90 fits across the two production configurations confirmed the use of aligned environmental rasters rather than the DIANWEI or final_env_for_maxent tables as environmental inputs. The latter intermediate table contains unexplained numerical values for the two records with missing source-raster values, so it was not used to reconstruct modeling inputs. This audit verifies the inspected extraction and fitting-input records but does not recover the complete historical 22-to-nine predictor-selection sequence.

Pearson correlation coefficients were calculated across the 7333 background grid cells with valid data for all nine environmental variables. At each iteration, the variable pair with the largest absolute correlation coefficient exceeding 0.8 was identified and the variable with the higher mean absolute correlation with all other variables in the current set was removed. This procedure continued until all absolute pairwise correlation coefficients among the retained variables were ≤0.8. The selection rule uses only predictor correlations and does not use species responses or AUC as selection criteria. The recorded removals were bio17, bio12, bio16, and bio14, in that order, leaving a maximum absolute pairwise correlation of 0.7923 (Tables S1 and S12). Five variables were retained: aspect (aspect), mean diurnal temperature range (bio02), precipitation of the wettest month (bio13), elevation (dem), and slope (slope) (Table S1). This configuration was used to assess sensitivity to variable combinations and does not represent an optimal predictor set identified through systematic optimization.

For the separate temperature-inclusive sensitivity analysis, we replaced static elevation (dem) in the five-variable set with annual mean temperature (bio01), retaining aspect, bio02, bio13, and slope. The maximum absolute Pearson correlation was 0.7542 across the current background and 0.7982 across the future input sets. BIO1 represents annual mean temperature, whereas BIO2 represents mean diurnal temperature range. This configuration is a predictor-configuration sensitivity analysis rather than an optimized model or an isolated temperature effect, because replacing dem with BIO1 changes both climate and terrain representation. The nine-variable production models and their reported results were retained unchanged (Supplementary Table S13).

Future climate data were obtained from the Coupled Model Intercomparison Project Phase 6 (CMIP6) bioclimatic datasets provided by WorldClim 2.1 for three global climate models: BCC-CSM2-MR, MIROC6, and MPI-ESM1-2-HR. For each model, two scenarios—SSP1-2.6 and SSP5-8.5—and two periods, the 2050s (2041–2060) and 2070s (2061–2080), were selected. The WorldClim future products used in the production analysis had a spatial resolution of 2.5 arc-minutes. The recovered records do not document why this product resolution was originally selected, so we treat it as the archived production baseline rather than an optimized choice. Future bioclimatic rasters were resampled to 30 arc-seconds using bilinear interpolation, with the current bio02 layer as the snap raster to standardize cell size, spatial extent, and grid alignment; this step did not increase the spatial information content of the 2.5-arc-minute products. The separately distributed WorldClim 30-arc-second CMIP6 layers evaluated in Table S17 are downscaled input products, not native GCM outputs; this paired diagnostic held the fitted models constant while substituting these inputs. Topographic variables were held constant for future periods, while climatic variables were replaced with data for the corresponding GCM, scenario, and period. Reference [35] describes the construction of the underlying WorldClim climate dataset; the CMIP6 models, scenarios, and periods listed here follow the metadata of the files used in this study. A catalog and endpoint check on 15 October 2026 confirmed the availability of all 12 selected 30-arc-second combinations (Table S17).

Elevation, slope, and aspect were held constant as topographic predictors. Predicted suitability can nevertheless change across elevations as climatic inputs change. These projections do not simulate actual dispersal or colonization, and static elevation does not explicitly represent future warming at a given elevation. BIO1 varies with the GCM, scenario, and period only in the separate temperature-inclusive diagnostic configuration.

For each food bamboo species, the MaxEnt models were projected onto four scenario–period combinations for BCC-CSM2-MR, MIROC6, and MPI-ESM1-2-HR: SSP1-2.6 in the 2050s, SSP1-2.6 in the 2070s, SSP5-8.5 in the 2050s, and SSP5-8.5 in the 2070s. For each species and scenario–period combination, the ten bootstrap projections were averaged separately for each GCM, yielding three continuous suitability rasters.

### 4.3. MaxEnt Model Development and Uncertainty Assessment

Species distribution models for the three food bamboo species were developed using MaxEnt 3.4.4 [36,37]. The regularization multiplier was set to 2; automatic feature selection was disabled; and linear, quadratic, and product (LQP) features were enabled, while hinge and threshold features were disabled. The features with nonzero coefficients retained in each bootstrap replicate were determined through model fitting and regularization. Models were run with 10 bootstrap replicates, with the random seed option enabled. Training samples were generated by sampling with replacement in each replicate; therefore, no fixed training-to-test split was specified.

To assess sensitivity to model complexity, we compared six configurations using the same occurrence records, environmental domain, and 7333 jointly valid background cells: linear features with RM = 2; linear plus quadratic features with RM = 2; LQP features with RM = 1, 2, or 4; and the five-variable LQP configuration with RM = 2. The four spatial folds used in the main validation were retained. For each configuration, we recorded test AUC, test omission, and the number of fitted feature coefficients with nonzero values. Zero-coefficient features and the four normalization and metadata entries in each lambda file were excluded from the count. Full-data diagnostic fits were projected to the 12 combinations of GCM, scenario, and period to summarize the range and direction of future area changes. This analysis was designed to quantify sensitivity rather than to select an optimal model, and the production ensemble used in the main text was not replaced.

The nine environmental variables shared 7333 valid grid cells, all of which were used as background cells. The output format was set to cloglog, and the final continuous suitability raster was calculated as the pixel-wise mean of predictions from the 10 replicates. Environmental variable percent contributions were summarized as arithmetic means across the 10 bootstrap replicates. AUC alone was not used to determine whether models were overfitted [34].

The original spatial four-fold validation used the archived species-specific occurrence partitions, with three groups used for training and one withheld for testing in each fold. These assignments were verified by reproducing them with the get.block function in the installed ENMeval 2.0.5.2 [38], using the lat_lon orientation. Each fold was fitted separately using the same LQP feature combination and RM of 2 as the main models, without bootstrapping within folds. The two variable configurations used identical occurrence-point partitions. In this original design, all 7333 jointly valid grid cells were used as the full-domain background in every fold; background cells were not withheld according to the spatial partitions. Model performance was evaluated using test AUC and test omission rate, with means and standard deviations reported across the four folds. The omission threshold for each fold was the 10th percentile of predicted values at that fold’s training points, calculated using the type = 7 algorithm. This procedure evaluated spatially withheld presence records from the existing dataset; it did not provide external validation using newly collected field records (Table S2).

To separate the effect of evaluation-background extent from refitting with spatially restricted backgrounds, we compared four designs while retaining the original occurrence partitions: A, the original fitted models evaluated against all 7333 background cells; B, the same models evaluated against the archived held-out background group; B_aligned, the same models evaluated against a cell-aligned held-out group; and C, diagnostic models fitted to three occurrence groups and their aligned background groups, evaluated using the withheld occurrence and background groups. The archived occurrence and background assignments were reproduced with the installed get.block algorithm; the historical calling script was unavailable. In *Y. brevipaniculata* and *C. szechuanensis*, one occupied grid cell per species had a center on the opposite side of a partition boundary from its occurrence coordinate. For the aligned diagnostics only, those two background cells were assigned to their occurrence group. No occurrence assignments or archived files were changed. Training and test backgrounds were disjoint in design C, and no test-occurrence grid cell was included in its training background (Table S11; Figure S3).

The 24 design-C fits retained the original LQP features, RM of 2, predictor values, cloglog output, and absence of within-fold bootstrapping. They used samples-with-data inputs, in which the predictor values were supplied for each occurrence and background location, while retaining the original MaxEnt sample-to-background setting. A full-domain control fit exactly reproduced the saved *B. faberi* nine-variable fold-1 occurrence and grid predictions. AUC was calculated from presence and background predictions at the same numeric precision with half credit for ties. Each fitted model used its own training-occurrence 10th percentile (type = 7) to count test omissions. Designs A, B, and B_aligned used identical models and thresholds and therefore identical omission counts. We reported fold counts, arithmetic mean fold metrics, and pooled omitted/test counts separately. These diagnostics evaluated background-design sensitivity without optimizing parameters or replacing production predictions.

For each species, configuration, and fold, we compared individual test-occurrence values with the corresponding training-occurrence and aligned training-background minima and maxima. We also summarized held-out background cells outside the training-background ranges. Raw aspect ranges were retained in the diagnostic table, but the count of records outside any marginal range used sine and cosine of aspect in place of raw degrees because aspect is circular. Within these coverage checks, the components were diagnostic quantities only; the original fitted models were unchanged. Separate terrain-representation refits are described below. A record outside any component range was counted once. The checks describe marginal coverage and do not test multivariate novelty or demonstrate a causal explanation of omission. Figure S3 shows the aligned background partitions and sample counts without precise occurrence locations; a restricted point-overlay diagnostic is retained for confidential evaluation subject to the data-sharing requirements.

We evaluated two supplementary aspect representations using the original spatial folds, all 7333 full-domain background cells, LQP features, RM = 2, and cloglog output. First, the existing model-grid angular aspect was replaced by cosine and sine components. Second, components were calculated from the archived fine-resolution aspect raster before bilinear interpolation to the original model-grid centers. For the second diagnostic, neighbors coded as −1 were excluded, remaining interpolation weights were renormalized, and the interpolated components were retained without unit-length normalization. Replacing one angular predictor with two components increased predictor counts from nine to ten and from five to six. Each aspect representation was evaluated for two base configurations, three species, and four folds, giving 24 fits per representation and 48 additional fits in total. These comparisons test representation sensitivity together with changes in available model features (Table S15).

For the slope audit, we calculated Horn gradients from complete 3 × 3 neighborhoods of the archived fine-resolution DEM and interpolated slopes to the model-grid centers using the corresponding four-cell bilinear weights. We compared a constant horizontal conversion of 111,000 m per degree with latitude-dependent east–west and north–south distances calculated from the WGS84 ellipsoid. The latter is a local differential-distance approximation, not a reproduction of the ArcGIS geodesic surface-fitting algorithm. We replaced only slope in 24 additional fits across the two original predictor configurations, three species, and four folds, retaining angular aspect and all other inputs and settings. Predictor counts remained nine and five. Original-input controls for the first *B. faberi* fold in each configuration reproduced the archived occurrence predictions and exported test AUCs. All terrain diagnostics used each fitted model’s training-occurrence 10th percentile (type = 7), counted test predictions strictly below that threshold as omissions, and reported arithmetic mean fold metrics separately from pooled omitted/test counts. These diagnostic fits did not replace the production ensembles (Table S15).

To assess how threshold choice affected suitable-area estimates and the direction of projected change, we compared the 10P threshold with fixed thresholds of 0.5 and 0.6. Predicted values at the retained valid occurrence points were extracted from the mean cloglog suitability raster generated from the 10 bootstrap predictions under current climatic conditions. The 10th percentile of these values was used as the species-specific 10P threshold for mapping, calculated using R’s default type = 7 quantile algorithm. The 10P thresholds for *Bashania faberi*, *Yushania brevipaniculata*, and *Chimonobambusa szechuanensis* were 0.3133, 0.2924, and 0.3166, respectively, when rounded for display; calculations retained the unrounded values documented in Tables S9 and S10. These species-specific thresholds were applied to binarize the current and future ensemble-mean suitability rasters (Table 8). These mapping thresholds were calculated from all retained valid occurrence points on the mean prediction raster and were distinct from the thresholds calculated separately from the training points in each spatial validation fold.

The fixed thresholds of 0.5 and 0.6 were also used to quantify areas where current and future ensemble-mean cloglog predictions reached the corresponding levels. The 10P threshold was used for the primary binary suitable-area calculations and landscape pattern analyses, whereas the fixed thresholds of 0.5 and 0.6 were used to assess whether the main conclusions regarding area change depended on a single threshold. These thresholds were used solely to binarize continuous predictions and do not represent habitat quality classes established through independent field validation. Comparing these thresholds allowed us to assess how threshold choice affected the extent and boundaries of predicted suitable areas [25,26,27].

For future predictions, the models fitted in the 10 bootstrap replicates under current climatic conditions were projected separately onto the environmental data for each GCM, emissions scenario, and period using density.Project with clamping enabled (doclamp = true) and cloglog output. For each species and each GCM–scenario–period combination, the arithmetic mean of the 10 projections was first calculated at each pixel to obtain the mean prediction for that GCM. The mean predictions from BCC-CSM2-MR, MIROC6, and MPI-ESM1-2-HR were then averaged with equal weights to generate the future ensemble-mean suitability raster. Pixel-wise sample standard deviations were also calculated across the three GCM mean predictions, using a denominator of 3 − 1, to characterize inter-GCM disagreement. These operations were performed using terra version 1.8-93 in R 4.5.2. The original analyses of future suitable area, threshold sensitivity, landscape metrics, and Schoener’s *D* used the ensemble-mean predictions; additional outcome-level GCM and bootstrap comparisons are described below. Inter-GCM standard deviations quantify variation among the selected climate-model projections, rather than total model uncertainty [23,24].

To ensure comparability between the two variable configurations, the sampled records and their multiplicities in each replicate were reconstructed from the sample prediction files of the original 10 bootstrap runs. Identical resampled records were then used for paired model fitting under the nine-variable and five-variable configurations. The paired nine-variable predictions reproduced the 30 original replicate outputs exactly; the maximum absolute difference between the resulting current or future ensemble means and the mean predictions used in the main analyses was less than 10^−6^. Both configurations used RM = 2 and LQP, retained duplicate records generated by sampling with replacement (removeduplicates = false), and used the same full-domain background, future climate inputs, and ensemble-averaging procedure. For each configuration, predicted values at valid presence points were extracted from its current mean raster across the 10 replicate predictions to calculate its own species-specific 10P thresholds. These thresholds were applied to the current and future predictions from the corresponding configuration. The fixed thresholds of 0.5 and 0.6 were identical across configurations. All area changes were calculated relative to the current area under the same variable configuration and threshold, and the directions of area change, landscape metrics, and Schoener’s *D* were compared between configurations (Tables S3–S5).

For the elevation-response diagnostic (Figure S5), we used the original ten fitted nine-variable models for *B. faberi* and *Y. brevipaniculata* without refitting. Each archived coefficient file was evaluated separately using its stored feature scaling, clamping, normalization, entropy, and cloglog transformation. We checked predictions against all 7333 valid cells of each original current raster, the 501 archived elevation-curve points per replicate, and the saved training-sample predictions; maximum absolute discrepancies were below 10^−6^. Replicate-specific conditioning values were taken from the archived training means and checked against the resampled records, preserving their multiplicities. We inspected nonzero elevation-containing terms and compared the archived fixed-mean curves with mean predictions under actual environmental combinations, weighting cells equally within 500 m elevation bands that included their lower boundary and excluded their upper boundary, and repeating the summaries with 250 m bands. Geographic predictions were averaged within each band for each replicate and then across replicates; sample SDs describe between-replicate variation, not confidence intervals. Occurrence and background-cell percentages and within-band precipitation ranges describe available environmental support rather than population frequencies corrected for survey effort. Source values and fitted terms are supplied with Figure S5; precise coordinates are excluded.

To propagate GCM differences to reported outcomes, we calculated suitable area, the six landscape metrics, and continuous-surface Schoener’s *D* separately from each GCM’s ten-bootstrap mean prediction under both predictor configurations. Suitable areas used geographic cell areas, and landscape metrics used the common 5442-cell, 1 km equal-area grid and eight-neighbor patch delineation described in Section 4.4. The species- and configuration-specific 10P thresholds were derived from current ensemble-mean predictions; the fixed thresholds remained 0.5 and 0.6. All thresholds were retained for the corresponding GCM-specific comparisons. For the combined resource, species were thresholded separately before taking their union within each GCM. Each future outcome was compared with the corresponding current ten-bootstrap-mean outcome under the same configuration and threshold. We reported individual GCM values; ranges; sample standard deviations; and counts of increase, decrease, unchanged, and undefined outcomes. We also compared the arithmetic mean of the GCM-specific outcomes with the outcome calculated from the three-GCM mean surface. If a metric was undefined for any GCM, the all-three-GCM mean was undefined; any mean over the remaining defined values was labeled separately with its sample count (Table S10).

Bootstrap variation in species-specific areas was assessed at all three thresholds, and variation in species-specific landscape metrics was assessed at 10P on the 1 km grid. For each species, a future bootstrap prediction was paired with the current prediction from the same fitted replicate; differences were summarized separately for each GCM using empirical ranges, medians, standard deviations, and direction counts. The 10P thresholds derived from current ensemble-mean predictions were not re-estimated for individual replicates; the fixed thresholds remained 0.5 and 0.6. Percentage changes were undefined when the paired current area was zero. For pairwise Schoener’s *D*, all 10 × 10 combinations of the two species’ bootstrap predictions were evaluated, with the same pair of model identities retained for each current–future comparison. These 100 combinations assume independent species resampling; they are not 100 independent observations or confidence intervals. Replicate numbers were not treated as paired across species, and no joint bootstrap distribution of combined-resource landscape metrics was estimated.

For spatial agreement, each GCM-specific mean prediction was thresholded and compared with the common current ten-bootstrap mean map. At each geographic cell, we counted the GCMs predicting a loss of current suitability or a gain of suitability. Combined-resource counts used the union of the three species within each GCM. Maps coded losses as −1 to −3 and gains as +1 to +3; zero indicated no projected threshold transition, and cells outside the common domain were retained as NoData. All three thresholds were evaluated; Figures S1 and S2 display the 10P results. Counts and associated geographic areas are reported in Table S10 and do not represent occurrence probabilities.

Environmental novelty was assessed separately for each GCM, scenario, period, and predictor configuration using MESS. The reference comprised the same 7333 jointly valid current environmental cells used as the modeling background. For each predictor, values within the reference range were assigned percentile-based similarity scores that increased toward the center of the reference distribution; values below or above that range received negative scores proportional to their distance beyond the corresponding range limit. The minimum score across predictors defined MESS. Cells with MESS < 0 were classified as strict range extrapolation, indicating that at least one predictor exceeded its calibration range. All summaries were restricted to the common valid domain, with cells outside it retained as NoData. Cell percentages used 7333 as the denominator; area-weighted summaries used the existing geodesic cell-area raster. We overlaid the GCM-specific novelty masks on changes derived from the production-matched bootstrap ensemble to distinguish areas within the shared current calibration ranges under all three GCM projections from areas with strict extrapolation in one or two GCMs or in all three. This was a spatial overlay, not MESS calculated from an averaged climate surface. A continuous MOP analysis was not performed (Table S8).

To quantify clamping sensitivity, the same full-data LQP RM = 2 model for each species and predictor configuration was projected with doclamp = true and doclamp = false to all 12 GCM–scenario–period combinations. Absolute differences in cloglog predictions were summarized over the common domain, including proportions of cells with differences >10^−6^, >10^−4^, and >10^−3^. These cutoffs describe numerical magnitudes and are not field-validated ecological thresholds. We also compared suitable areas using each full-data model’s own 10th-percentile training-presence threshold and fixed thresholds of 0.5 and 0.6. These deterministic full-data comparisons were supplementary transfer diagnostics; they did not replace the ten bootstrap production predictions, and their thresholds and areas were kept separate from the main ensemble analysis (Table S8).

### 4.4. Landscape Metrics and Structural Continuity Analysis

Binary suitable/unsuitable maps were generated for each species under current and future conditions using the 10P threshold to quantify the landscape structure of predicted suitable areas. Suitable area was calculated from the original 30-arc-second binary rasters using the actual area of each valid grid cell. Before calculating landscape metrics, the binary rasters were reprojected to the ESRI:102025 equal-area coordinate system using nearest-neighbor resampling and standardized to a 1 km × 1 km grid. The common valid-data mask contained 5442 cells (5442 km^2^) and was identical across species, predictor configurations, thresholds, and scenarios. Spatially contiguous suitable patches were identified using the eight-neighbor rule for the baseline analysis. The R package landscapemetrics version 2.2.1 [39] was used to calculate six class-level metrics for the suitable class: percentage of landscape (PLAND), number of patches (NP), largest patch index (LPI), mean patch area (AREA_MN), mean Euclidean nearest-neighbor distance (ENN_MN), and aggregation index (AI). AI uses shared-edge like adjacencies and does not depend on the four- or eight-neighbor choice for patch delineation.

Because the future climate layers originated at 2.5 arc-minutes and were resampled to 30 arc-seconds for projection, we assessed sensitivity to the spatial grain of the outputs. Continuous current and future ensemble predictions were aggregated in 5 × 5 geographic-cell blocks to a common 2.5 arc-minute grid using arithmetic means of valid cells. After thresholding, binary predictions were projected by nearest-neighbor resampling to a fixed ESRI:102025 grid with 5 km cells. Its analysis mask was generated by projecting the original 30-arc-second valid-domain mask directly to that template, rather than extending the domain to all aggregation blocks containing any valid cells. The 5 km mask contained 225 cells (5625 km^2^) and was identical across all comparisons at that grain. The original geographic domain contained 7333 valid cells with a cell-area-based total of 5434.81 km^2^; its representations on the 1 km and 5 km equal-area grids differed because of grid discretization. This analysis aggregated predictions after fitting and did not refit models using harmonized climate predictors.

To assess input-product sensitivity separately from post-prediction aggregation, we checked the WorldClim CMIP6 30-arc-second catalog on 15 October 2026 and confirmed the listings and HTTP availability of all 12 selected GCM–scenario–period combinations. Regional subsets of the official 30-arc-second products were cropped onto the existing geographic model grid, whose cell centers and resolution aligned without interpolation. We retained the original 90 fitted models across three predictor configurations, three species, and ten bootstrap repetitions, and generated 1080 new future projections using unchanged coefficients, cloglog output, clamping, terrain layers, and thresholds. The baseline inputs already had a 30-arc-second grid; no new occurrence records or model refitting were required for this paired projection test. Each GCM prediction was averaged across ten repetitions, and future ensembles were equal-weight means of the three GCM maps. Areas were calculated over the same 7333 geographic cells, and landscape metrics used the same 5442-cell 1 km equal-area mask and eight-neighbor patch delineation. Species and combined-resource outcomes were compared at 10P, 0.5, and 0.6. This tests the choice of distributed future climate product at fixed fitted models, not a pure interpolation-kernel effect or independent predictive accuracy (Table S17).

The sensitivity design crossed the nine- and five-variable configurations, 1 km and 5 km grains, species-specific 10P and fixed 0.5 and 0.6 thresholds, and four- and eight-neighbor patch definitions. It used the production ensemble surfaces: ten-bootstrap means for current predictions and equal-weight means of three GCM-specific ten-bootstrap means for future predictions. The 10P values were calculated from current 30-arc-second ensemble predictions at the final occurrence coordinates using R quantile type 7 and held fixed for all scenarios and both grains within each species–configuration combination. Combined suitable area was defined as the logical union of the three separately thresholded species maps. Six metrics were calculated for the three species and their union under the current baseline and four future scenario–period combinations, giving 480 combinations. Changes were evaluated against the corresponding current result with the same configuration, grain, threshold, and patch rule (Tables S7 and S9).

Where no suitable cells remained, suitable area, PLAND, NP, and LPI were recorded as zero, whereas AREA_MN, ENN_MN, and AI were recorded as undefined. ENN_MN was also undefined when only one patch remained. AI was undefined for a single suitable cell because its maximum possible like adjacency was zero; multiple isolated suitable cells could instead yield a defined AI of zero. Undefined values were retained explicitly in the comparisons and were not replaced with zeros.

The binary suitability maps for the three food bamboo species were summed pixel by pixel to generate a suitable bamboo species richness layer. Pixels where at least one species met its species-specific 10P threshold were also combined to create a combined suitable-area layer representing potential bamboo resources, for which landscape metrics were calculated using the same procedure. Because suitable-area estimates and landscape metrics were derived from different spatial grids, slight differences may occur between areas calculated from the original rasters and patch-area metrics calculated on the 1 km equal-area grid. These metrics characterize the spatial structure and structural continuity of predicted suitable areas; they do not measure functional connectivity for giant panda movement. Patch attributes were included to complement suitable-area estimates by providing additional information on changes in the spatial structure of potential resources [28].

For the elevation-band analysis, cells in the common 7333-cell geographic modeling domain were assigned to fixed 500 m intervals from 1000 to <5000 m using the modeling DEM. Intervals included their lower boundary and excluded their upper boundary. Cell-area-weighted summaries were calculated for each species, all three predictor configurations, all three thresholds, and the four future scenario–period combinations, with individual GCMs and ensemble-mean predictions reported separately. Current maps were ten-bootstrap means, and each future GCM map was its corresponding ten-bootstrap mean. The archived configuration-specific 10P thresholds and fixed thresholds of 0.5 and 0.6 were retained. Gross loss comprised currently suitable cells becoming unsuitable; gross gain comprised currently unsuitable cells becoming suitable; net change equaled gain minus loss. Percentage changes were undefined when the baseline suitable area was zero. For *C. szechuanensis*, unanimous GCM loss required all three GCM-specific maps to fall below the threshold at a cell classified as currently suitable under the same configuration. We intersected these loss masks between configurations and across the four scenario–period combinations. The 500 m bands are descriptive strata, not inferred ecological boundaries. This analysis reused existing predictions and did not refit models or estimate new bootstrap intervals (Table S16; Figure S4).

Schoener’s *D* was used to assess spatial overlap between the continuous suitability surfaces of different food bamboo species [40]. For current conditions, the mean continuous suitability raster from the 10 bootstrap predictions was used for each species; for future conditions, the three-GCM ensemble-mean continuous suitability raster was used for each corresponding scenario in the primary analysis. The additional comparisons used GCM-specific ten-bootstrap means and the bootstrap combinations described in Section 4.3. Calculations were restricted to pixels with valid predictions for both species. The shared geographic-grid cells were weighted equally, rather than by cell area. Within this shared set of pixels, each species’ continuous suitability values were divided by their sum to obtain *pi* and *qi*, and *D* was calculated as

$$D=1-\frac{1}{2}\sum_{i}\left|p_{i}-q_{i}\right|$$

*D* ranges from 0 to 1, with larger values indicating greater spatial overlap between the two continuous suitability surfaces. This metric characterizes changes in spatial relationships among the predicted suitable distributions of food bamboo species across climate scenarios; it does not represent actual coexistence among bamboo species, dietary substitution, or functional connectivity for giant pandas.

## 5. Conclusions

Under common MaxEnt settings, the three food bamboo species differed in environmental associations and current potential distributions. These differences support the species-specific assessment of projected climate responses to inform regional bamboo monitoring and conservation within the modeled Qionglai Mountains study area.

Future suitable-area changes depended on GCM, scenario, threshold, predictor configuration, aggregation order, and future climate product. The nine-variable 10P ensemble projected late-century SSP5-8.5 expansion for *Bashania faberi* and *Yushania brevipaniculata*, but each expansion was supported by only one of the three GCMs and reversed under the five-variable ensemble or higher fixed thresholds. *Chimonobambusa szechuanensis* contracted across the original two production ensemble configurations, thresholds, and scenarios, with exceptions in the temperature-inclusive temp5 diagnostic and among individual GCMs. Ensemble-map summaries therefore do not imply unanimous climate-model responses.

The additional BIO1-containing diagnostic confirms that predictor choice can change the projected direction and the combined spatial pattern. It should be read as a temperature-inclusive configuration comparison: replacing static elevation with BIO1 changes the predictor set, while the original nine-variable ensemble remains the production baseline.

For the ensemble-mean predictions using the original future climate products, combined PLAND and AI declined in every future scenario under the original two production configurations and all three thresholds on the 1 km grid, with declining AREA_MN under both patch definitions. These declines also held at 10P on the 5 km grid, but higher coarse-grid thresholds and separate GCM outcomes produced exceptions. Patch number and pairwise overlap did not change uniformly. The results support monitoring suitable extent, patch configuration, and interspecific spatial relationships while retaining explicit model and processing uncertainty. Small occurrence samples and conditional model assumptions limit site-specific inference. In particular, the *B. faberi* projections based on 15 records are exploratory, and its landscape metrics are indicative; they should not be used as precise estimates of occupied habitat or site-level management outcomes. The projections do not directly quantify actual food supply, colonization, or functional connectivity for giant pandas. Independent records and resource-quality assessments remain necessary. The official 30-arc-second product comparison retained species-level 10P area directions but changed some higher-threshold and landscape outcomes, including the sign of the small late-century nine-variable AI change. Climate-product choice must therefore accompany the other uncertainty qualifications.

## Figures and Tables

**Table 1:** Spatial four-fold cross-validation results for MaxEnt models of the three food bamboo species under the nine-variable baseline configuration.

Species	Test Points per Fold	Test AUC, Mean ± SD	Test AUC Range	Mean Test Omission Rate
*Bashania faberi*	3–4	0.9308 ± 0.0727	0.8581–0.9985	37.50%
*Yushania brevipaniculata*	6–7	0.8593 ± 0.1218	0.6833–0.9608	20.83%
*Chimonobambusa szechuanensis*	10–11	0.9083 ± 0.0614	0.8176–0.9538	37.05%

Note: Models used linear, quadratic, and product (LQP) features with a regularization multiplier (RM) of 2. Means and standard deviations were calculated across four spatial validation folds. In each fold, presence points from three spatial groups were used for training, and those from the withheld group were used for testing. For the original validation summarized here, all 7333 jointly valid grid cells across the full study domain were used as background in every fold. Test omission rates were calculated using the 10th percentile of predicted values at the training points in each fold as the threshold; the reported values are the arithmetic means of omission rates across the four folds. This validation used existing occurrence records and does not constitute external validation with newly collected field data. AUC, area under the receiver operating characteristic curve; SD, standard deviation. Standard deviations of test omission rates are provided in Table S2; additional background-partition comparisons are reported separately in Table S11.

**Table 2:** Current and future suitable areas based on species-specific 10P thresholds (km^2^).

Species	Current	SSP1-2.6 2050s	SSP1-2.6 2070s	SSP5-8.5 2050s	SSP5-8.5 2070s
*Bashania faberi*	435.83	81.81	59.71	244.82	472.72
*Yushania brevipaniculata*	1052.92	706.45	738.93	975.55	1119.12
*Chimonobambusa szechuanensis*	607.55	507.76	558.37	475.00	434.08

Note: Current areas were derived from mean predictions across 10 bootstrap replicates. Future areas were derived from equally weighted ensemble means of three global climate models (GCMs), each based on the mean of 10 replicate projections. 10P, 10th-percentile presence threshold; SSP, Shared Socioeconomic Pathway. The 2050s and 2070s represent 2041–2060 and 2061–2080, respectively.

**Table 3:** Suitable areas of the three food bamboo species under different thresholds (km^2^).

Species	Threshold	Current	SSP1-2.6 2050s	SSP1-2.6 2070s	SSP5-8.5 2050s	SSP5-8.5 2070s
*Bashania faberi*	10P	435.83	81.81	59.71	244.82	472.72
*Bashania faberi*	0.5	232.25	0.74	0.00	2.95	0.00
*Bashania faberi*	0.6	164.38	0.00	0.00	0.00	0.00
*Yushania brevipaniculata*	10P	1052.92	706.45	738.93	975.55	1119.12
*Yushania brevipaniculata*	0.5	531.38	241.12	218.31	356.96	341.24
*Yushania brevipaniculata*	0.6	389.36	105.70	71.73	139.11	112.42
*Chimonobambusa szechuanensis*	10P	607.55	507.76	558.37	475.00	434.08
*Chimonobambusa szechuanensis*	0.5	354.49	268.08	299.36	236.80	204.77
*Chimonobambusa szechuanensis*	0.6	255.44	176.49	206.29	157.11	133.29

**Table 4:** Landscape metrics for single-species suitable areas.

Species	Scenario	PLAND (%)	NP	LPI (%)	AREA_MN (ha)	ENN_MN (m)	AI (%)
*Bashania faberi*	Current	7.96	5	5.66	8660.00	2236.07	75.12
*Bashania faberi*	SSP1-2.6 2050s	1.47	4	0.70	2000.00	2000.00	64.08
*Bashania faberi*	SSP1-2.6 2070s	1.10	5	0.53	1200.00	2094.43	57.69
*Bashania faberi*	SSP5-8.5 2050s	4.45	5	3.60	4840.00	3069.48	72.79
*Bashania faberi*	SSP5-8.5 2070s	8.78	4	8.62	11,950.00	2575.14	81.47
*Yushania brevipaniculata*	Current	19.66	79	4.13	1354.43	2523.23	56.85
*Yushania brevipaniculata*	SSP1-2.6 2050s	12.94	105	1.23	670.48	2520.81	43.80
*Yushania brevipaniculata*	SSP1-2.6 2070s	13.69	100	1.08	745.00	2409.33	43.41
*Yushania brevipaniculata*	SSP5-8.5 2050s	18.03	93	1.69	1054.84	2407.41	50.82
*Yushania brevipaniculata*	SSP5-8.5 2070s	20.75	94	2.43	1201.06	2442.60	52.83
*Chimonobambusa szechuanensis*	Current	11.14	54	6.17	1122.22	2741.69	61.02
*Chimonobambusa szechuanensis*	SSP1-2.6 2050s	9.41	48	3.56	1066.67	2628.67	61.96
*Chimonobambusa szechuanensis*	SSP1-2.6 2070s	10.42	52	4.13	1090.38	2744.77	61.97
*Chimonobambusa szechuanensis*	SSP5-8.5 2050s	8.82	49	3.36	979.59	2663.64	60.04
*Chimonobambusa szechuanensis*	SSP5-8.5 2070s	8.03	46	3.11	950.00	2580.41	56.97

Note: Landscape metrics were calculated from binary suitability maps generated using species-specific 10P thresholds, with the ESRI:102025 equal-area projection, a 1 km grid, and the eight-neighbor rule for patch delineation. PLAND, percentage of landscape (%); NP, number of patches; LPI, largest patch index (%); AREA_MN, mean patch area (ha); ENN_MN, mean Euclidean nearest-neighbor distance (m); AI, aggregation index (%). SSP denotes Shared Socioeconomic Pathway and 10P denotes the 10th-percentile presence threshold. AI was calculated from shared-edge like adjacencies, independently of the patch-delineation rule. The common landscape mask contained 5442 valid 1 km cells (5442 km^2^).

**Table 5:** Landscape metrics for the combined suitable area of the three food bamboo species.

Scenario	PLAND (%)	NP	LPI (%)	AREA_MN (ha)	ENN_MN (m)	AI (%)
Current	34.20	81	6.17	2297.53	2248.03	65.03
SSP1-2.6 2050s	21.00	92	3.60	1242.39	2357.78	55.32
SSP1-2.6 2070s	21.15	86	4.17	1338.37	2249.01	55.73
SSP5-8.5 2050s	25.56	76	3.80	1830.26	2306.80	60.25
SSP5-8.5 2070s	31.05	80	10.11	2112.50	2263.88	63.72

Note: The combined suitable area comprises pixels where at least one food bamboo species meets its species-specific 10th-percentile presence (10P) threshold. PLAND, percentage of landscape (%); NP, number of patches; LPI, largest patch index (%); AREA_MN, mean patch area (ha); ENN_MN, mean Euclidean nearest-neighbor distance (m); AI, aggregation index (%). SSP denotes Shared Socioeconomic Pathway.

**Table 6:** Pairwise Schoener’s *D* values among bamboo species under different climate scenarios.

Species Pair	Current	SSP1-2.6 2050s	SSP1-2.6 2070s	SSP5-8.5 2050s	SSP5-8.5 2070s
*B. faberi*–*Y. brevipaniculata*	0.3027	0.2824	0.2387	0.2447	0.3105
*B. faberi*–*C. szechuanensis*	0.0273	0.0288	0.0287	0.0325	0.0383
*Y. brevipaniculata*–*C. szechuanensis*	0.3288	0.4146	0.4830	0.4806	0.4444

Note: Schoener’s *D* was calculated from continuous suitability surfaces; three-GCM ensemble-mean continuous suitability maps were used for future scenarios.

**Table 7:** DEM-derived elevation summaries for the final modeling records of the three food bamboo species.

Species (*n*)	DEM-Derived Elevation (m), Min–Median–Max	Relative Position Among Final Records
*Bashania faberi* (*n* = 15)	1995–2811–3215	Higher-elevation records
*Yushania brevipaniculata* (*n* = 25)	1670–2136–2587	Intermediate-elevation records
*Chimonobambusa szechuanensis* (*n* = 43)	1265–1664–2191	Lower-elevation records

Note: Elevations were extracted from the DEM at the final modeling occurrence locations. They are raster-derived values, not field measurements, and describe the retained modeling records rather than the full elevational limits of each species.

**Table 8:** Threshold settings for the three food bamboo species.

Species	10P Threshold	Fixed Threshold 1	Fixed Threshold 2
*Bashania faberi*	0.3133	0.5	0.6
*Yushania brevipaniculata*	0.2924	0.5	0.6
*Chimonobambusa szechuanensis*	0.3166	0.5	0.6

## Data Availability

Non-sensitive data supporting the findings are provided in the article and its Supplementary Materials, including Supplementary Dataset S6, Tables S1–S17, Figures S1–S5, and the Figure S5 source-data archive. These materials provide non-sensitive predictor values, model settings, validation results, and numerical data underlying the tables and figures. The analysis-code archive provides a script index, configuration templates for verified replay workflows, and documentation of external or restricted inputs. Figure 9 displays final occurrence locations as map symbols; the Supplementary Materials do not include a machine-readable coordinate table. Raw field records and exact occurrence coordinates are not publicly deposited because they are subject to data-sharing restrictions associated with Giant Panda National Park and conservation considerations. Restricted records and source-level audit materials may be provided to the editorial office for confidential evaluation where required and permitted by applicable data-sharing requirements. Restricted inputs are not part of the public code archive.

## Supplementary Materials

The following supporting information can be downloaded at: https://www.mdpi.com/article/10.3390/plants15213408/s1, Supplementary Dataset S6: Occurrence record identifiers, non-sensitive environmental predictor values, point-processing and model-setting summaries, validation results, and numerical data from the original production analysis. Supplementary Dataset S6 and Supplementary Table S6 are separate files; additional revision diagnostics are provided in Tables S6–S17. Table S1: Environmental variable correlations and selection of the low-correlation predictor set; Table S2: Spatial four-fold validation summaries and fold-level results; Table S3: Paired comparisons of suitable-area changes between the nine-variable and five-variable configurations; Table S4: Landscape metrics under the two predictor configurations; Table S5: Pairwise Schoener’s *D* under the two predictor configurations. Table S6: Model-complexity sensitivity analysis, including cross-validation metrics and full-data future-projection diagnostics; Table S7: Landscape output-grain sensitivity using masks derived from the original valid domain; Table S8: Environmental novelty, clamping sensitivity, and spatial overlap between environmental extrapolation and ensemble suitability changes; Table S9: Landscape sensitivity across predictor configurations, spatial grains, thresholds, and patch-delineation rules, including undefined-value conventions and valid-grid metadata; Table S10: GCM-specific outcomes, aggregation-order comparisons, paired species bootstrap changes, pairwise overlap sensitivity, and spatial agreement summaries; Table S11: Original and geographically partitioned background validation, fold counts, environmental-range diagnostics, and alignment and reproduction checks; Table S12: Recovered predictor-provenance records, DIANWEI extraction and missing-value diagnostics, final-point and fitting-input checks, and the distinction between historical evidence and new diagnostics; Table S13: BIO1-containing predictor-configuration sensitivity analysis, including validation, suitable-area, individual-GCM, combined-landscape, and univariate BIO1-range summaries; Table S14: Marginal environmental predictor ranges at the final occurrence records and comparisons of environmental support before and after record processing; Table S15: Terrain-processing evidence, numerical slope reconstruction, and aspect and slope validation diagnostics; Table S16: Elevation-band suitable-area transitions, *C. szechuanensis* loss agreement, cross-configuration intersections, and candidate monitoring strata; Table S17: Paired comparison of the original 2.5-arc-minute and official 30-arc-second future climate products, including availability and source metadata, climate-input reconstruction, and area and landscape sensitivity at fixed fitted models. Figure S1: Spatial GCM agreement in suitability changes under the nine-variable 10P configuration. Figure S2: Corresponding agreement under the five-variable configuration. Figure S3: Aligned spatial background partitions and occurrence counts for the three bamboo species; precise occurrence locations are not shown. Figure S4: GCM agreement on *C. szechuanensis* losses under all three predictor configurations. Tables S12–S15 exclude precise occurrence coordinates. Figure S5 provides the elevation-response diagnostic, occurrence and background elevation summaries, and precipitation-support comparisons for the two species with declining marginal elevation responses. Figure_S5_source_data.zip contains the underlying numerical summaries and model terms; the corresponding scripts are indexed in the analysis-code collection. Analysis_Code_Revision_20261020.zip: Indexed analysis scripts, configuration templates for verified replay workflows, and documentation of required external and restricted inputs. Execution requires local path configuration and access to the inputs specified for each workflow.
