# Supplementary material for: Climate-Change Projections of Habitat Suitability and Spatial Patterns for Three Giant Panda Food Bamboos in the Qionglai Mountains Region, China
Source: Plants (Basel). 2026 Nov 2;15(21):3408. doi: 10.3390/plants15213408 (PMC13765571; doi:10.3390/plants15213408)
Supplement: Supplementary file 1 [file plants-15-03408-s001.zip › Supplementary_Materials_Revision/Supplementary_Figures/Figure_S2.pdf]

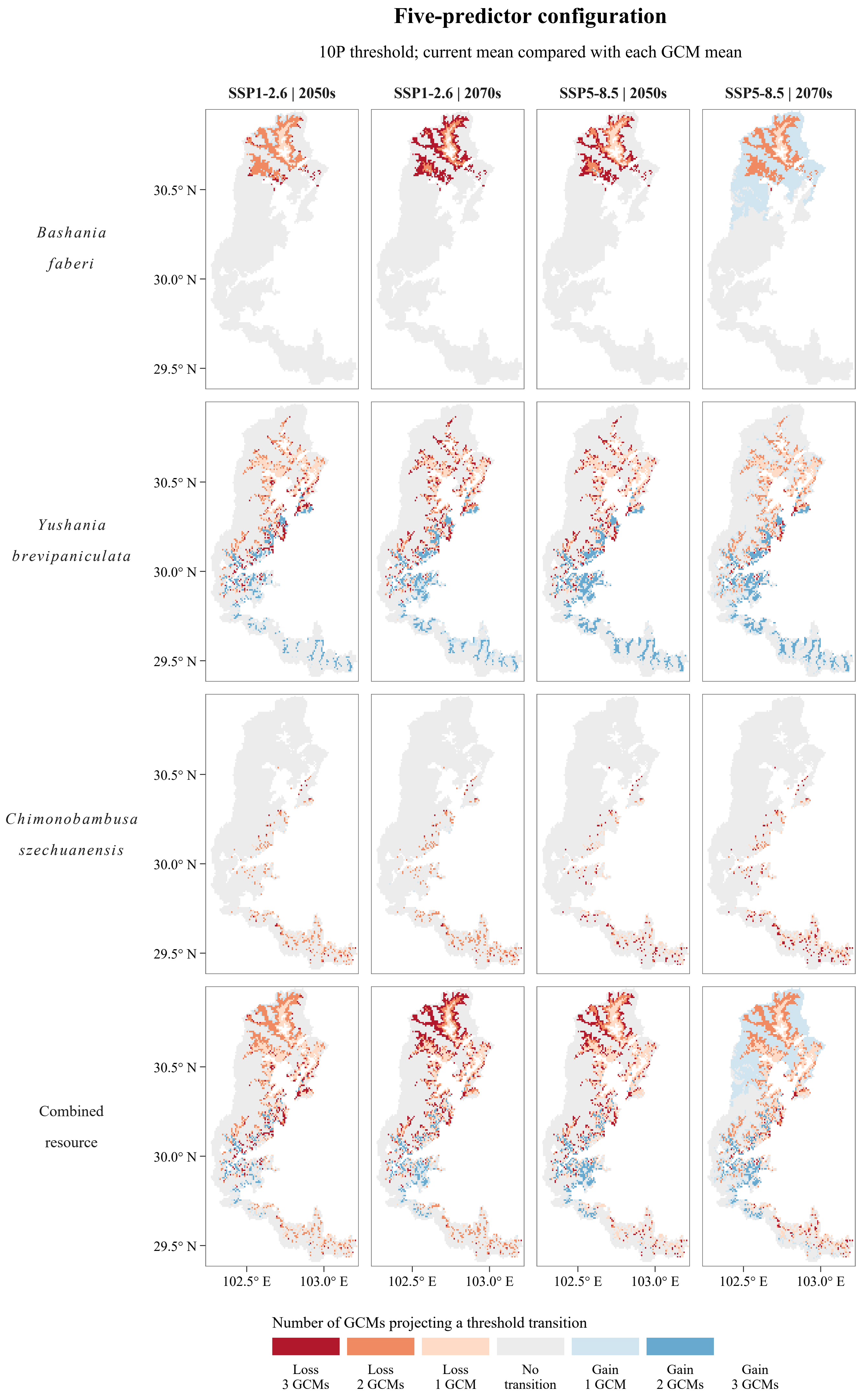

Five-predictor configuration
10P threshold; current mean compared with each GCM mean
SSP1-2.6 | 2050s
SSP1-2.6 | 2070s
SSP5-8.5 | 2050s
SSP5-8.5 | 2070s
Bashania faberi
Yushania brevipaniculata
Chimonobambusa szechuanensis
Combined resource
30.5° N
30.0° N
29.5° N
102.5° E
103.0° E
Number of GCMs projecting a threshold transition
Loss 3 GCMs
Loss 2 GCMs
Loss 1 GCM
No transition
Gain 1 GCM
Gain 2 GCMs
Gain 3 GCMs
