# Supplementary material for: Climate-Change Projections of Habitat Suitability and Spatial Patterns for Three Giant Panda Food Bamboos in the Qionglai Mountains Region, China
Source: Plants (Basel). 2026 Nov 2;15(21):3408. doi: 10.3390/plants15213408 (PMC13765571; doi:10.3390/plants15213408)
Supplement: Supplementary file 1 [file plants-15-03408-s001.zip › Supplementary_Materials_Revision/Supplementary_Figures/Figure_S3.pdf]

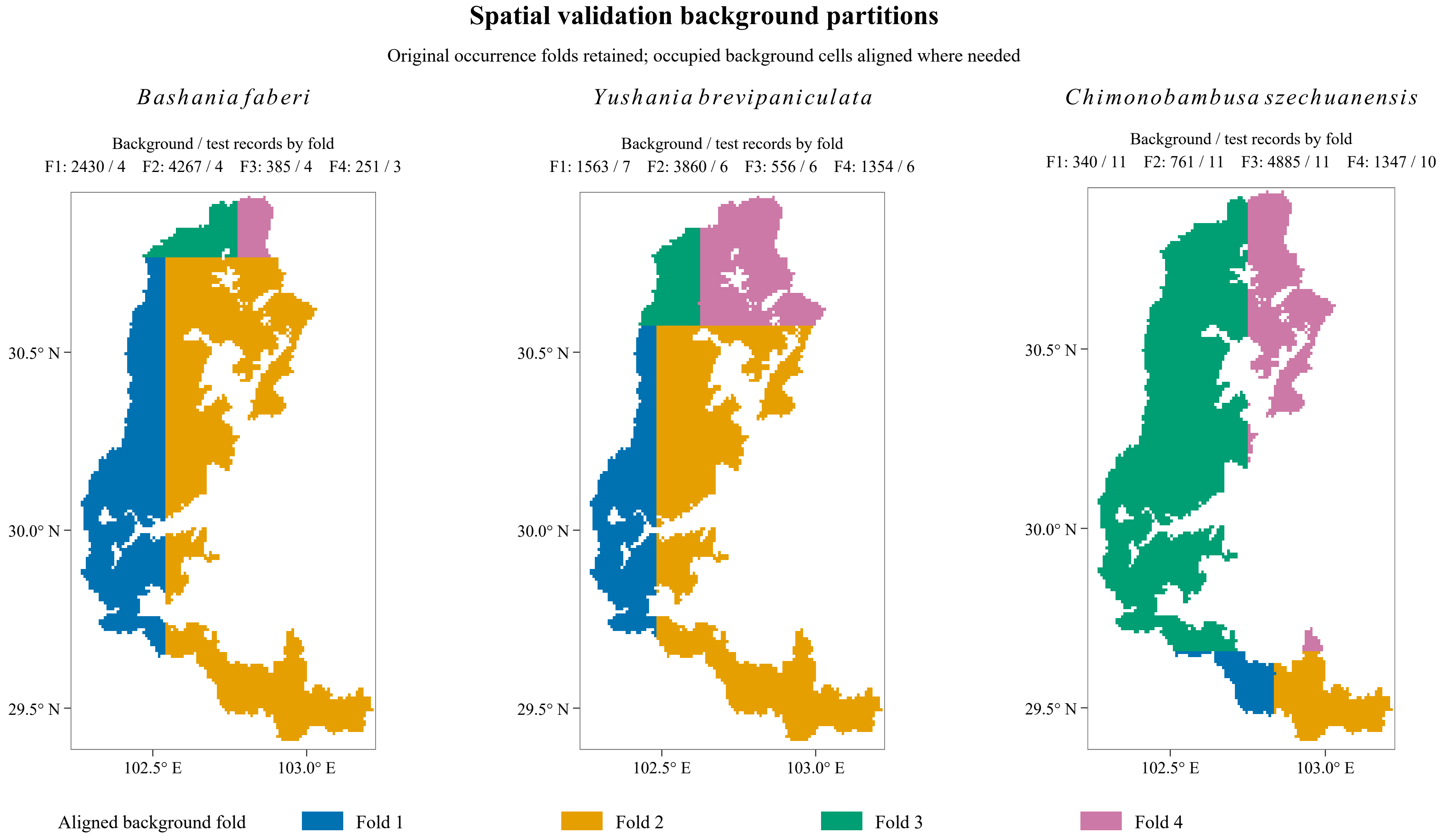

Spatial validation background partitions
Original occurrence folds retained; occupied background cells aligned where needed
Bashania faberi
Background / test records by fold
F1: 2430 / 4 F2: 4267 / 4 F3: 385 / 4 F4: 251 / 3
Yushania brevipaniculata
Background / test records by fold
F1: 1563 / 7 F2: 3860 / 6 F3: 556 / 6 F4: 1354 / 6
Chimonobambusa szechuanensis
Background / test records by fold
F1: 340 / 11 F2: 761 / 11 F3: 4885 / 11 F4: 1347 / 10
30.5° N
30.0° N
29.5° N
102.5° E
103.0° E
Aligned background fold
Fold 1
Fold 2
Fold 3
Fold 4
