# Supplementary material for: Climate-Change Projections of Habitat Suitability and Spatial Patterns for Three Giant Panda Food Bamboos in the Qionglai Mountains Region, China
Source: Plants (Basel). 2026 Nov 2;15(21):3408. doi: 10.3390/plants15213408 (PMC13765571; doi:10.3390/plants15213408)
Supplement: Supplementary file 1 [file plants-15-03408-s001.zip › Supplementary_Materials_Revision/Supplementary_Figures/Figure_S4.pdf]

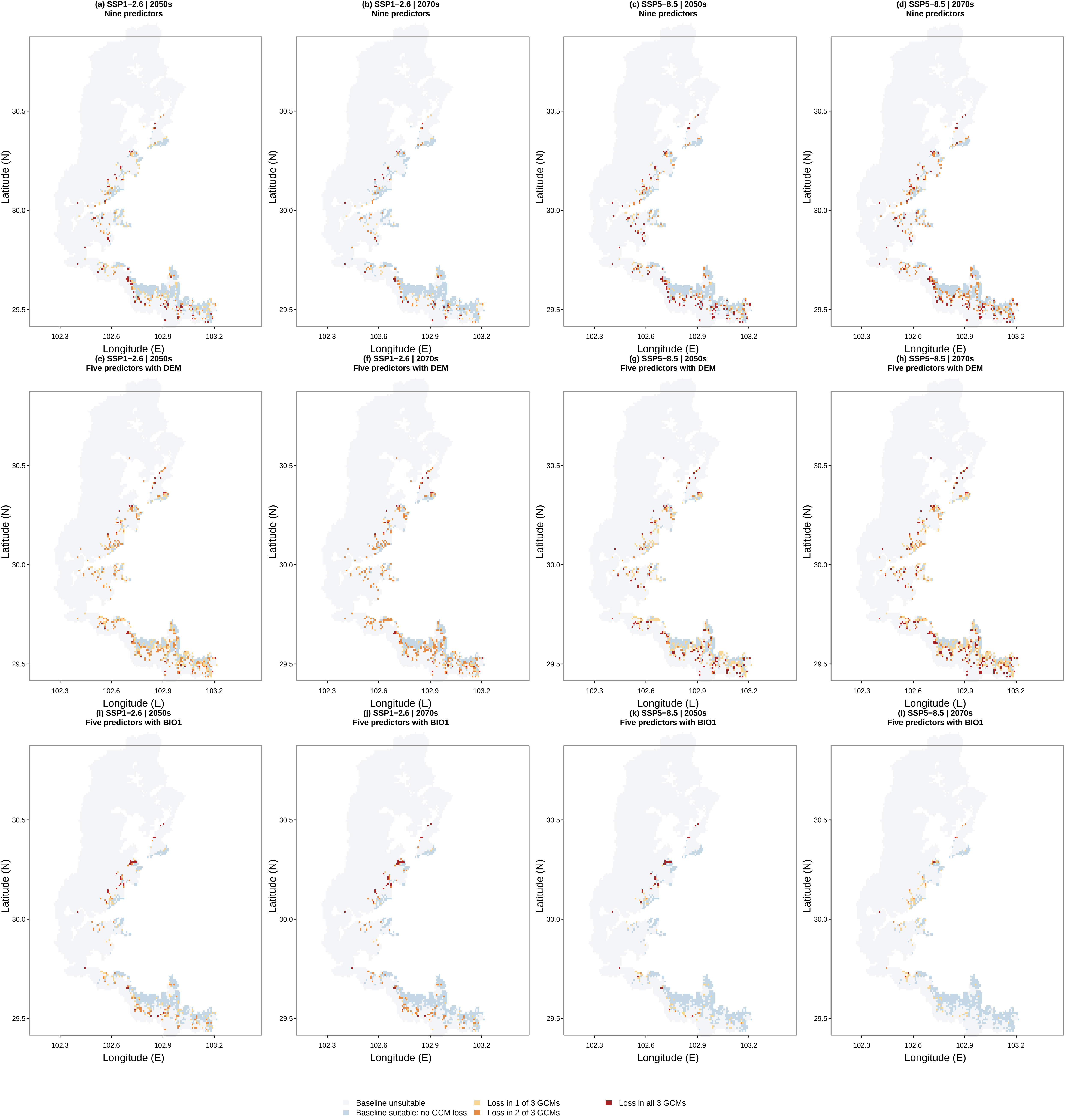

(a) SSP1−2.6 | 2050s
Nine predictors
(b) SSP1−2.6 | 2070s
Nine predictors
(c) SSP5−8.5 | 2050s
Nine predictors
(d) SSP5−8.5 | 2070s
Nine predictors
(e) SSP1−2.6 | 2050s
Five predictors with DEM
(f) SSP1−2.6 | 2070s
Five predictors with DEM
(g) SSP5−8.5 | 2050s
Five predictors with DEM
(h) SSP5−8.5 | 2070s
Five predictors with DEM
(i) SSP1−2.6 | 2050s
Five predictors with BIO1
(j) SSP1−2.6 | 2070s
Five predictors with BIO1
(k) SSP5−8.5 | 2050s
Five predictors with BIO1
(l) SSP5−8.5 | 2070s
Five predictors with BIO1
Latitude (N)
30.5
30.0
29.5
Longitude (E)
102.3
102.6
102.9
103.2
Baseline unsuitable
Baseline suitable: no GCM loss
Loss in 1 of 3 GCMs
Loss in 2 of 3 GCMs
Loss in all 3 GCMs
