# Supplementary material for: Climate-Change Projections of Habitat Suitability and Spatial Patterns for Three Giant Panda Food Bamboos in the Qionglai Mountains Region, China
Source: Plants (Basel). 2026 Nov 2;15(21):3408. doi: 10.3390/plants15213408 (PMC13765571; doi:10.3390/plants15213408)
Supplement: Supplementary file 1 [file plants-15-03408-s001.zip › Supplementary_Materials_Revision/Supplementary_Figures/Figure_S5.pdf]

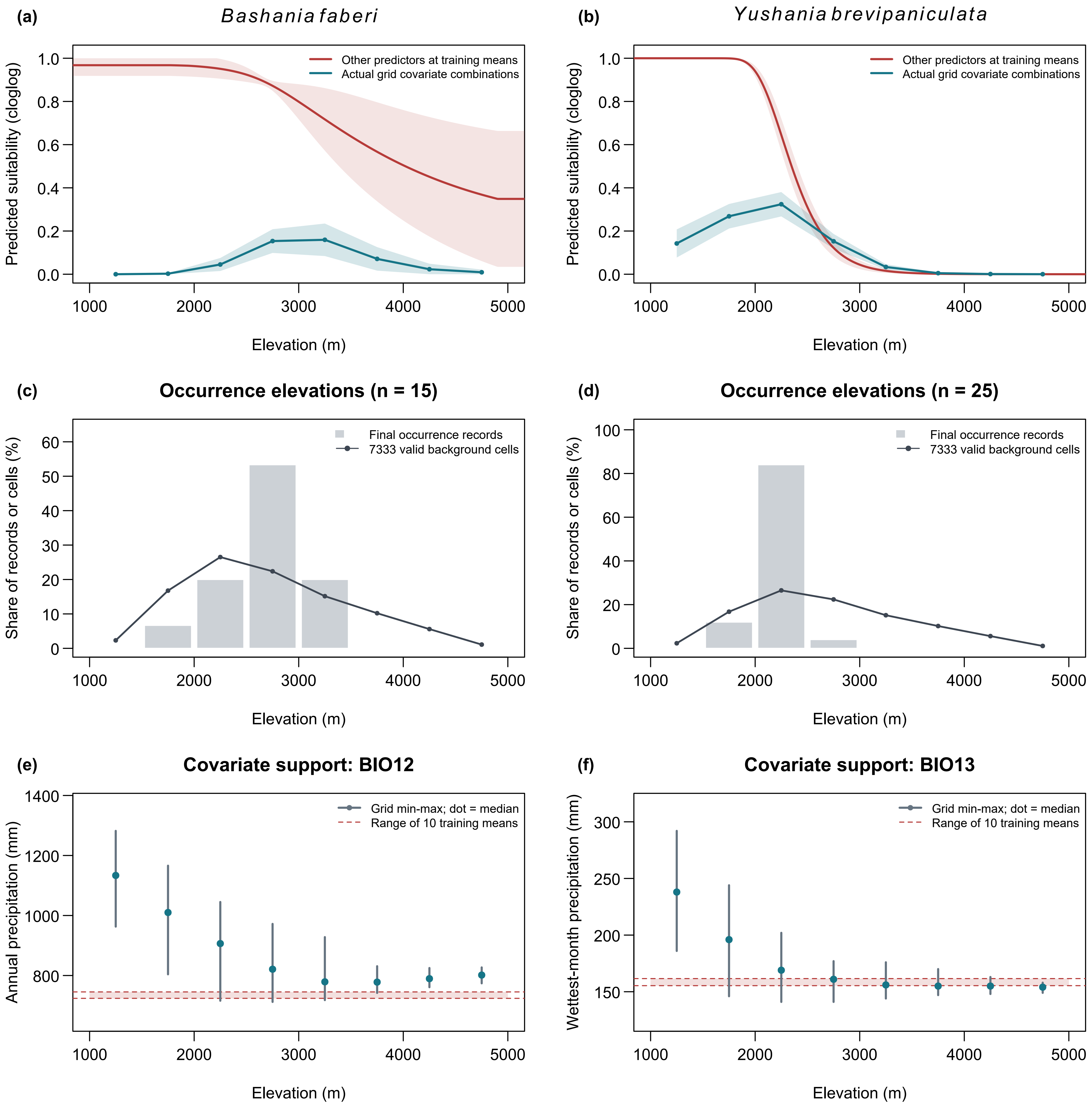
(a)
Bashania faberi
Other predictors at training means
Actual grid covariate combinations
Predicted suitability (cloglog)
Elevation (m)
(b)
Yushania brevipaniculata
Other predictors at training means
Actual grid covariate combinations
Predicted suitability (cloglog)
Elevation (m)
(c)
Occurrence elevations (n = 15)
Final occurrence records
7333 valid background cells
Share of records or cells (%)
Elevation (m)
(d)
Occurrence elevations (n = 25)
Final occurrence records
7333 valid background cells
Share of records or cells (%)
Elevation (m)
(e)
Covariate support: BIO12
Grid min-max; dot = median
Range of 10 training means
Annual precipitation (mm)
Elevation (m)
(f)
Covariate support: BIO13
Grid min-max; dot = median
Range of 10 training means
Wettest-month precipitation (mm)
Elevation (m)
